# Privacy-Preserving Federated Survival Support Vector Machines for Cross-Institutional Time-To-Event Analysis: Algorithm Development and Validation

**DOI:** 10.2196/47652

**Published:** 2024-03-29

**Authors:** Julian Späth, Zeno Sewald, Niklas Probul, Magali Berland, Mathieu Almeida, Nicolas Pons, Emmanuelle Le Chatelier, Pere Ginès, Cristina Solé, Adrià Juanola, Josch Pauling, Jan Baumbach

**Affiliations:** 1 Institute for Computational Systems Biology University of Hamburg Hamburg Germany; 2 LipiTUM, Chair of Experimental Bioinformatics TUM School of Life Sciences Technical University of Munich Freising Germany; 3 MetaGenoPolis INRAE Université Paris-Saclay Jouy-en-Josas France; 4 Liver Unit Hospital Clínic de Barcelona Barcelona Spain; 5 Institut d’Investigacions Biomèdiques August Pi i Sunyer (IDIBAPS) Barcelona Spain; 6 Centro de Investigacion en Red de Enfermedades hepaticas y Digestivas (CIBEReHD) Madrid Spain; 7 Faculty of Medicine and Health Sciences University of Barcelona Barcelona Spain

**Keywords:** federated learning, survival analysis, support vector machine, machine learning, federated, algorithm, survival, FeatureCloud, predict, predictive, prediction, predictions, Implementation science, Implementation, centralized model, privacy regulation

## Abstract

**Background:**

Central collection of distributed medical patient data is problematic due to strict privacy regulations. Especially in clinical environments, such as clinical time-to-event studies, large sample sizes are critical but usually not available at a single institution. It has been shown recently that federated learning, combined with privacy-enhancing technologies, is an excellent and privacy-preserving alternative to data sharing.

**Objective:**

This study aims to develop and validate a privacy-preserving, federated survival support vector machine (SVM) and make it accessible for researchers to perform cross-institutional time-to-event analyses.

**Methods:**

We extended the survival SVM algorithm to be applicable in federated environments. We further implemented it as a FeatureCloud app, enabling it to run in the federated infrastructure provided by the FeatureCloud platform. Finally, we evaluated our algorithm on 3 benchmark data sets, a large sample size synthetic data set, and a real-world microbiome data set and compared the results to the corresponding central method.

**Results:**

Our federated survival SVM produces highly similar results to the centralized model on all data sets. The maximal difference between the model weights of the central model and the federated model was only 0.001, and the mean difference over all data sets was 0.0002. We further show that by including more data in the analysis through federated learning, predictions are more accurate even in the presence of site-dependent batch effects.

**Conclusions:**

The federated survival SVM extends the palette of federated time-to-event analysis methods by a robust machine learning approach. To our knowledge, the implemented FeatureCloud app is the first publicly available implementation of a federated survival SVM, is freely accessible for all kinds of researchers, and can be directly used within the FeatureCloud platform.

## Introduction

Accessing data to apply machine learning (ML) in biomedical settings is still challenging [1]. Large amounts of data exist in clinical settings but are scattered across numerous institutions. Due to strict privacy regulations, such as the General Data Protection Regulation (GDPR), this data cannot be easily shared or collected at a central institution [2]. This causes hurdles for cross-institutional biomedical analyses that depend on highly sensitive patient data. One example is time-to-event analysis, aiming to find parameters that prolong or shorten the time until a particular event, such as death, occurs [3]. In these studies, the event of interest does not necessarily occur for all samples, increasing the need for large sample sizes [4]. Until today, the need for large sample sizes and heterogeneous data for time-to-event studies is still mainly solved through traditional data sharing, leading to the central collection of various deidentified and anonymized data sets from different centers. Since using anonymized data in the training of ML models tends to weaken model performance [5], this comes with a tradeoff of data privacy and data quality, accelerating the need for alternative methods that keep data private and ensure the quality of the data [6].

In recent years, federated learning (FL) has become a feasible alternative to central data collection by enabling the training of models on distributed data sets. Instead of sharing sensitive data with a central institution, in FL, only insensitive model parameters are shared with a central aggregation server [7,8]. Therefore, each participating party calculates its own model with local model parameters on their local data. These local model parameters are then shared with the aggregator and aggregated into a global model. Afterward, the global model is shared again with each participant and can be updated in another iteration. The first and probably most widely used aggregation approach is the federated average [9], calculating the weighted mean of the exchanged model parameters. Besides using different aggregation approaches, FL can also be distinguished between horizontal and vertical learning, as well as cross-device and cross-silo learning. Horizontal learning describes FL on data with the same features but different samples, while vertical learning performs on the same samples but with different features between the participating parties. Cross-device FL trains models across millions of participants (such as mobile phones), cross-silo FL, on the other hand, focuses on a few clients only, such as hospitals or research institutes [10].

Especially in combination with privacy-enhancing techniques (PETs), model parameters can be exchanged securely, such that a global aggregator or potential attacker cannot even see the local parameters of each participant [11]. This secure exchange of model parameters is necessary to comply with the GDPR, as even local models can be considered personal data [12]. Therefore, FL enables the training on a significantly larger data set compared with single-institution scenarios. While federated algorithms still often struggle with communication efficiency, the significantly increased amount of data can offset this performance issue, making FL a serious competitor to classical ML. Additionally, since FL models are trained on a larger variety of data, they typically generalize better than traditional ML models and even generalize faster in some cases [13,14]. Many FL approaches are already published for biomedical applications, such as medical imaging analysis, genome-wide association studies, or gene expression analysis [15-17].

In addition to federated ML approaches, several federated time-to-event analysis algorithms have been introduced recently and confirmed their high potential for privacy-preserving analyses [18-21]. However, existing approaches solely cover traditional statistical methods such as the estimation of survival functions and the Cox proportional hazards model. Modern ML algorithms for survival analysis, such as survival Support Vector Machines (SVMs), are not yet available in a federated fashion, even though SVMs belong to one of the most popular ML methods. If algorithms are not available in federated scenarios, this might be a reason why researchers chose not to perform FL, if their favorite algorithms are not available. Many well-performing centralized algorithms are challenging to translate to a federated scenario while keeping sensitive data private. Another limitation of FL is communication efficiency. FL algorithms need to exchange the intermediate statistics with a central aggregator, which is especially inefficient for algorithms with many iterations. This inefficiency even increases when adding secure aggregation schemes, such as additive secret sharing. This PET ensures that only masked and encrypted model parameters are shared with the aggregating party, securing the local models from data leakage [18].

To address the lack of availability of federated time-to-event methods, we propose a privacy-preserving, horizontally federated, cross-silo survival SVM based on the survival analysis package *scikit-survival* [22]. Compared with other existing time-to-event methods, such as the Cox proportional hazard model, the survival SVM allows an actual prediction of the time until an event happens. It can be used to predict the risk of individual samples, which is not possible in univariate time-to-event algorithms and is not the aim of the Cox proportional hazards model. Therefore, to the best of our knowledge, it is the first freely available federated survival prediction method. We implemented the algorithm as an app in the FeatureCloud platform to make it publicly accessible and to minimize the hurdles of FL infrastructure [23]. Based on a combination of FL and additive secret sharing, we show on 3 benchmark data sets, that our approach achieves highly similar results compared with central data analysis. Additionally, we apply it to a set of real-world microbiome data sets to demonstrate its applicability to original clinical data.

## Methods

Here, we propose the adapted algorithm for the federated survival SVM, describe its implementation as a FeatureCloud app, and explain how we evaluated its performance.

### Federated Survival SVM

**Figure 1:**
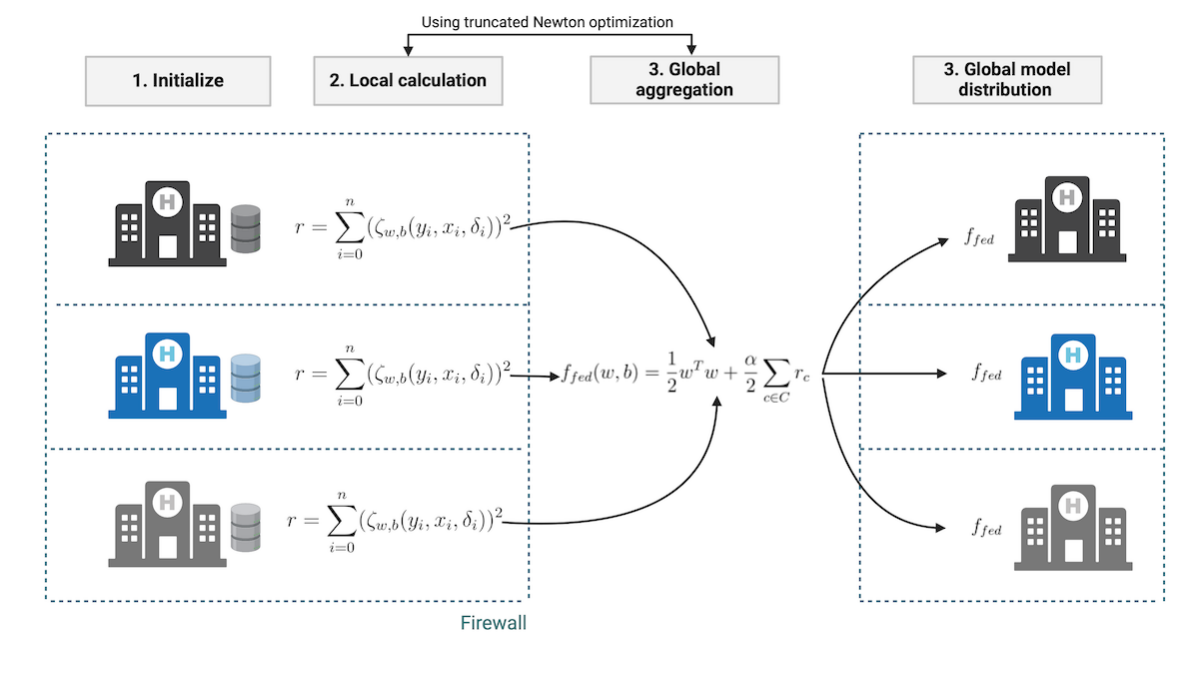
Federated calculation of a survival support vector machine (SVM). Each site calculates the sum of squares locally and sends it to the global aggregation server. The aggregation server aggregates the local sum of squares by summing them up to the global sum of squares. The objective function is minimized in a federated fashion by a truncated Newton approach. After convergence, the global model is distributed to all participating clients.

We extended the regression objective of *scikit-survival*’s FastSurvivalSVM without ranking to be applicable in federated environments [24]. As shown in Figure 1, instead of calculating the sum of the squared ζ-function centrally, it is calculated at each site, with the feature vector *x_i_*, the survival time *y_i_*>0, and the binary event indicator *δ_i_*. Each site’s local sum of squared ζ-function is then sent to a global aggregator and summed up to the global sum of squared ζ-function. The below equations show the central objective function and our corresponding federated objective function, with C being the set of all participating clients.



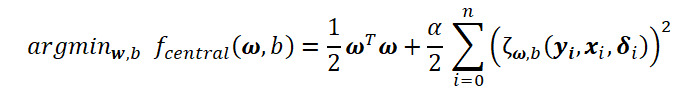





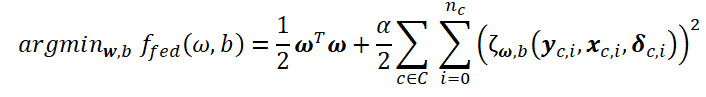





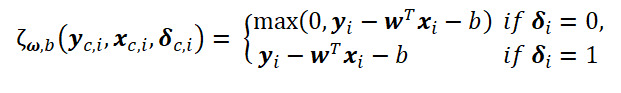



Mathematically, our federated formula leads to the same solution as the centralized calculation of the objective function. Similar to the centralized analysis, a truncated Newton method (such as Newton-CG) can be used to optimize the objective function. For this, in each iteration, the gradient and Hessian matrix of each client are also sent to the global aggregator to sum them up to the global gradient and Hessian matrix. To reduce potential privacy leakage from the exchanged data, the implementation of the federated algorithm should support a secure aggregation scheme that hides the locally exchanged data from attackers or the global aggregation server.

### FeatureCloud

We developed an FL app on the FeatureCloud platform to make our approach publicly available. To develop this app, we used the app template and application programming interface provided by FeatureCloud [25]. Using the *scikit-survival* package and Python, we implemented our algorithm, put it into the FeatureCloud app template, and published it in the FeatureCloud artificial intelligence store. It can be used with other apps in a workflow or standalone using the platform. Our code is entirely open source.

In FeatureCloud, 1 participating client also takes the aggregating role and is called the coordinator. The app is implemented as a state machine, meaning that the app switches between states to perform different tasks. All states and their transitions are shown in Multimedia Appendix 1. After reading the local data and config files, minimizing the objective function using a federated Newton conjugate gradient is performed iteratively. Therefore, the local gradient and Hessian matrices are calculated and sent to the coordinator. The coordinator aggregates these data to obtain the global matrices, updates the weight vector *ω*, and broadcasts it to all clients. This is repeated until convergence.

A considerable advantage of the FeatureCloud platform is its native support of 2 very popular PETs, such as secure multiparty computation (SMPC). For applying SMPC, FeatureCloud supports a secure aggregation scheme for hiding locally exchanged parameters using additive secret sharing [26]. Through this, the exchanged local models are protected, and only the global aggregations are visible to attackers, clients, and the global aggregator. This is achieved by splitting the value that needs to be exchanged with the global aggregator into *n* shards, where *n* is the number of participating clients, and the sum of these *n* shards would result in the actual value [23]. Each shard is encrypted using a public key of each participant. These encrypted shards are shared with the global aggregator, sending them to the corresponding client holding the private key. The clients decrypt the received shards, sum them up, and send them back to the global aggregator, which sums up all received sums. This final sum results in the actual, nonhidden, global aggregate.

### Ethical Considerations

According to German regulations, for our retrospective study performed on publicly available data or data with explicit consent, approval from an ethical committee was not required.

#### Evaluation

We evaluated our approach using the developed FeatureCloud app on 3 benchmark data sets, all available via the *scikit-survival* package. The breast cancer data set (BRCA) [27] contains the gene expression profiling of microarray experiments from 198 primary breast tumors, originally used to validate a 76-gene prognostic signature able to predict distant metastases in lymph node–negative patients with breast cancer. The German Breast Cancer Study Group 2 data set (GBSG2) [28] contains data from a multicenter randomized clinical trial to compare the effectiveness of 3 versus 6 cycles of cyclophosphamide, methotrexate, and fluorouracil on recurrence-free and overall survival of 686 women. The observed parameters were hormonal therapy (yes or no), age of the patients, menopausal status (pre vs post), tumor size (in mm), tumor grade, number of positive tumor nodes, progesterone receptor (in fmol), and estrogen, as well as the censoring indicator and recurrence-free survival time (in days). The Worcester Heart Attack Study data set (WHAS500) [29] contains data from 500 patients with acute myocardial infarction, collected during thirteen 1-year periods. Parameters were age, gender, initial heart rate, initial systolic and diastolic blood pressure, body mass index, history of cardiovascular disease, atrial fibrillation, cardiogenic shock, congestive heart complications, complete heart block, myocardial infarction order and type, vital status, and total length of follow-up.

Additionally, we evaluated our algorithm on a recent, high-dimensional gut microbiome data set from the Hospital Clinic of Barcelona, containing data from 150 patients with liver cirrhosis [30]. The data set was aimed at assessing the predicting role of the gut microbiome for the survival of the patients in the context of liver cirrhosis, using shotgun metagenomic sequencing performed on fecal DNA isolated from stool samples. A former version of the data has been previously analyzed with a different methodology [30]. For this study, the Metagenomic Species Pangenome (MSP) was used to identify and quantify microbial species associated with the IGC2 reference catalog [31]. MSPs are clusters of coabundant genes (minimum size >100 genes) used as a proxy for microbial species, reconstructed from 1601 metagenomes to 1990 MSP species [32]. MSP abundances were estimated as the mean abundance of their 100 marker genes, as far as at least 20% of these genes are detected. The MSP abundance table was then normalized in each sample by dividing its abundance by the sum of MSP abundances detected in the sample. Further details regarding the data sets are shown in Table 1.

**Table 1:** Overview of all data sets. Our 4 evaluation data sets differ greatly in the number of samples, features, events, and censored individuals. Features indicate the number of clinical variables or microbial species abundance in the data set; median follow-up indicates the median follow-up time of the patients in days; events indicate the number of patients for whom the event of interest was observed during observation time; and censored indicates the number of patients for whom the event of interest was not observed during observation time.

Data set	Samples, n	Features, n	Median follow-up (days)	Events, n	Censored, n	End point
BRCA	198	84	4384.0	51	147	Presence of metastases
GBSG2	686	11	1084.0	299	387	Recurrence-free survival
WHAS500	500	16	631.5	215	285	Death
Microbiome	150	1995	416.0	51	99	Death

^a^BRCA: breast cancer data set.

^b^GBSG2: German Breast Cancer Study Group 2 data set.

^c^WHAS500: Worcester Heart Attack Study data set.

We one-hot encoded nonbinary categorical features. For each data set, we created either 1 client (100%) as the centralized scenario, 3 clients (20%, 50%, and 30%) as the multicentric imbalanced scenario, and 5 clients (20% each) as the multicentric balanced scenario, and we split the data accordingly.

To evaluate the accuracy of our model, we used the Harrell concordance index, which was developed as a generalization of the area under the receiver operating characteristic curve for time-to-event models [33]. It corresponds to the probability of concordance between observed and predicted survival based on each pair of individuals. A c-index of 0.5 means that the model performs as well as a random guess, and a c-index of 1.0 means that the model predicts perfectly well.

After preprocessing, we performed a 3 × 3-fold cross-validation (CV) for a FeatureCloud workflow consisting of a federated normalization, the federated survival SVM, and a federated survival evaluation (c-index). We then compared our results with the centralized analysis of every client and the merged data set (simulating a central data collection). Centralized analysis was performed using *scikit-survival*’s FastSurvivalSVM with a rank ratio of 0, α of 0.0001, true fit intercept, and a maximum of 50 iterations. The same hyperparameters were used for the federated analysis, respectively.

#### Privacy

FeatureCloud supports several properties to increase the privacy and security of the computations. One important step is that FL projects can be only executed with invited participants. For this, a unique and secret code is needed to join the project. Every participant can see the workflow and each individually executed FeatureCloud app that will run in the workflow. As FeatureCloud apps are open source, even the executed code of the apps can be examined.

The execution of apps and workflows in FeatureCloud is containerized and strictly monitored. Due to the containerization, individual apps are not allowed to establish a connection to the internet, which prevents the extraction of data from malicious code. Even though the communication between clients does not contain sensitive patient information, it is RSA (Rivest–Shamir–Adleman) encrypted through the standard HTTPS protocol. This prevents unauthorized third parties from gaining insights into parameters exchanged during training.

Exchanged parameters from each individual site are masked through the secure aggregation scheme, hiding the intermediate statistics from other participating clients and the global aggregator. This efficiently addresses the problem of local models considered as personal data in GDPR [18].

Our federated survival SVM app currently uses a hybrid approach of SMPC and FL. This hybrid approach increases the privacy of the exchanged local parameters from both participants and potential attackers, as explained in the methods section.

Differential privacy (DP) [34] is not yet supported by FeatureCloud but is currently in development and could be added to the algorithm as an additional layer to improve privacy. However, as the app trains a linear model, it is less prone to overfit, reducing the surface for potential membership and attribute inference attacks [35]. In DP, noise is added to the model parameters during the training process to guarantee a mathematically quantifiable amount of privacy for each sample. While this comes with large advantages regarding privacy, the application of DP has also various weaknesses. The addition of noise lowers the performance of the model significantly, especially when applying the amount of noise necessary for a meaningful level of privacy [36]. Further, this guarantee only is applicable for a limited number of interactions with the resulting model. As the final model is distributed to all participants, they can interact with the model arbitrarily, making the privacy guarantee void, thus not warranting an inclusion in this analysis.

A PET not supported by FeatureCloud currently is homomorphic encryption (HE), which allows the computation of the model on encrypted values, making sharing of data even more secure. While this is great in theory, it actually gains very little benefit in this analysis scenario. The data we share is already nonsensitive and through the use of SMPC, we can hide not only the data but the data’s origin. This is why FeatureCloud currently supports SMPC instead of HE.

Our implementation of the federated survival SVM app uses all the functionalities offered by FeatureCloud and does not deviate from these best practices.

## Results

### Performance

**Figure 2:**
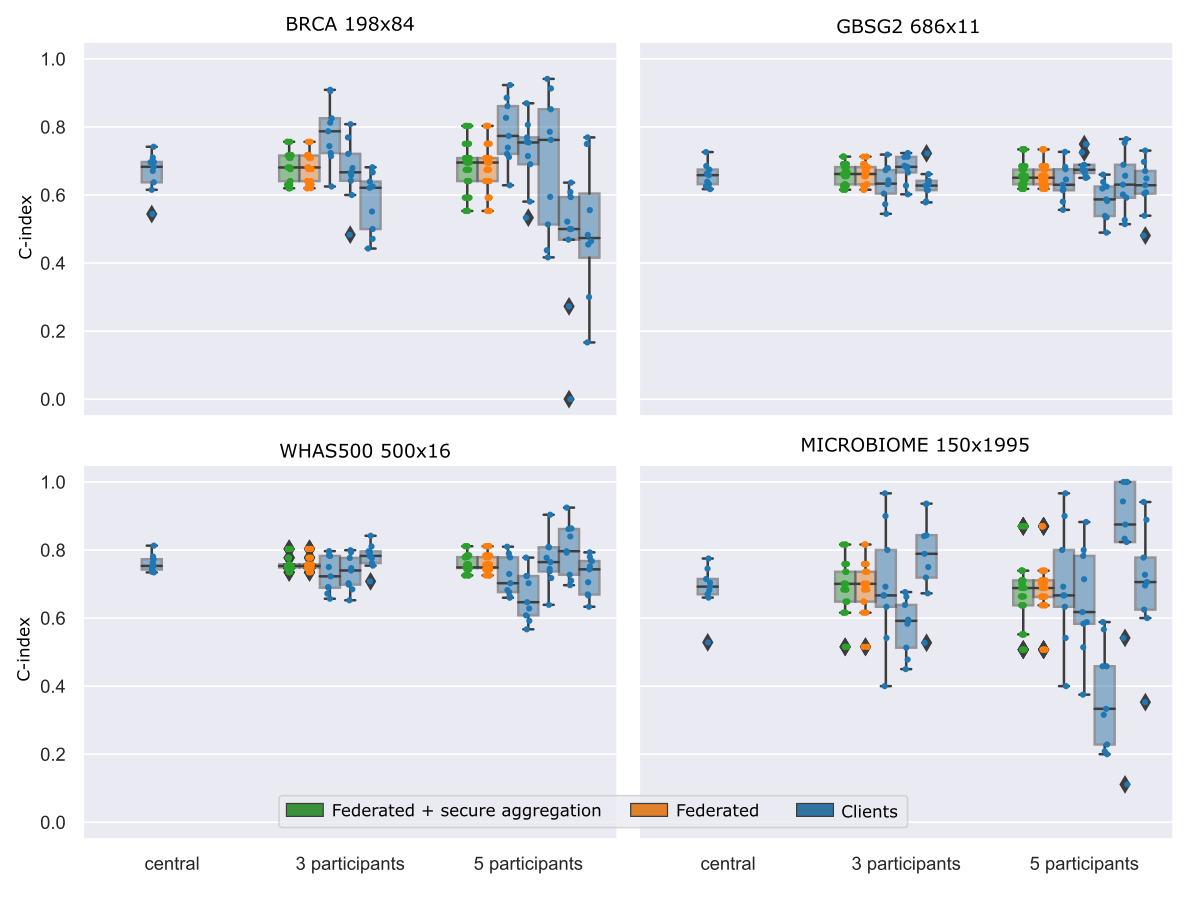
Comparison of federated and centralized analysis. The boxplots show the evaluated c-indices (3 × 3-fold cross validation) of the central, 3 participants, and 5 participants analysis (rows). For each scenario, we compared the federated and secure aggregation approach (green), the federated-only approach (orange), and the performance of every single site (blue). BRCA: breast cancer data set; GBSG2: German Breast Cancer Study Group 2 data set; WHAS500: Worcester Heart Attack Study data set.

Our workflow delivered a highly similar model performance and model parameters for all federated analyses compared with the ones performed on the corresponding centralized data sets. The resulting c-indices to estimate the performance of our time-to-event models are depicted in Figure 2 [33]. For each data set (subplot), we show a boxplot consisting of the evaluated c-index for each CV split of our federated workflow with secure aggregation (green), federated workflow without secure aggregation (orange), and centralized calculation for each individual client (blue). The CV results show that our federated as well as the federated and secure aggregation approach perform highly similar to the centralized estimates. The calculation of the federated c-index in FeatureCloud causes small deviances in the c-index between centralized and federated. This is because FeatureCloud calculates a local c-index and aggregates to the mean c-indices of all sites. Therefore, it does not lead to the same c-index as a central computation would. The mean c-indices for the 4 data sets are in the range between 0.658 (GSBG2) and 0.76 (WHAS500). In contrast to the accuracy, achieving very high c-indices is rather difficult and depends very much on the problem. In a bioinformatics context, the lowest c-index of 0.658 (GBSG2) can be considered as moderate. The model achieves discrimination between individuals with different survival outcomes. However, it might not be of clinical utility and needs further refinement. The c-index of 0.76 (WHAS500) on the other hand, can be considered as good and has predictive value. Improving the predictive value of the models and increasing c-index was out of the scope of this work. A complete table of the results is available in Multimedia Appendix 2.

The model weights are nearly identical, with a maximum difference of only 0.001 and a mean difference of 0.0002 (Multimedia Appendices 1 and 3). These tiny differences between the weights of the central model and our model are negligible, as they do not change the overall prediction results and still lead to equal c-indices. The resulting model is therefore almost identical to the one that was trained on central data. A useful property of the linear survival SVM is, that the model weights can be used as a feature importance measure, which is also supported in our approach.

Besides calculating the feature importance from model weights directly, our federated survival SVM app uses Shapley additive explanations (SHAP), an explainable artificial intelligence framework for the interpretation of ML models [37]. Using SHAP, we compared the final models of the central, federated without secure aggregation, and federated with secure aggregation runs. For each data set, the SHAP shows highly similar model interpretations with a mean Pearson correlation of 0.991 between the central and the federated model without secure aggregation, and a mean Pearson correlation of 0.985 between the central model and the federated model with secure aggregation. A slightly worse correlation in the secure aggregation model is expected, as the masking of local parameters leads to floating-point issues. The worst correlation is shown in the microbiome data set (0.964), which can be explained by the high correlation between features in this data set. The results of the SHAP correlation analysis are listed in Multimedia Appendix 4 and the corresponding SHAP beeswarm plots are available in Multimedia Appendix 5.

Our results further demonstrate the importance of large data sets, as the performance of the locally trained models on single clients (smaller sample size) shows a much higher variance than our federated models. If 5 institutes combine their small data sets, they can perform a much more reliable time-to-event analysis compared with isolated institutions. This further supports the high practical value of FL in real-world clinical time-to-event analysis, especially for institutions with small sample sizes, homogenous cohorts, or only a few patients with rare diseases.

### Runtime

**Figure 3:**
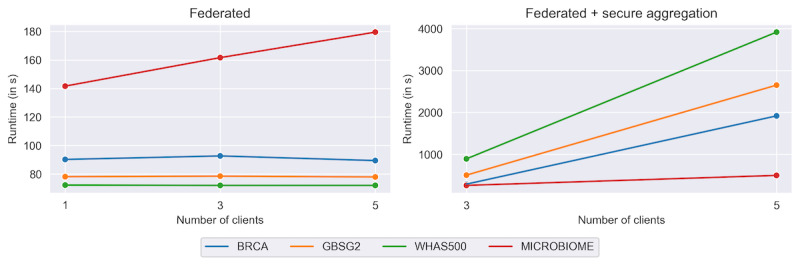
Runtime analysis. The lines represent the runtime for each data set and the number of participating clients. The federated-only approach is depicted on the left, and the federated and secure aggregation approach is depicted on the right.

As shown in Figure 3, the runtime largely depends on the data set. In the case of FL, the number of iterations and, therefore, the number of data exchanges are the bottleneck. While the federated-only approach has linear runtime, the runtime of federated and secure aggregation is much worse and increases with an increasing number of clients. As described in the FeatureCloud publication, providing better privacy by hiding the exchanged parameters from the global aggregator, the simple additive secret sharing grows quadratic with the number of participants. Especially when many iterations and data exchanges are needed, this has a bad influence on the runtime of the FL implementation.

All results of the runtime analysis are shown in Multimedia Appendix 6. Additionally, we performed the runtime analysis on a data set with a large sample size. As real-world time-to-event data sets are difficult to find, we used a synthetically generated, published data set from an example colon data set with 15,564 samples [38]. Our results show that our method scales well for large sample sizes, as the number of iterations is the bottleneck in FL (Multimedia Appendix 7).

### FeatureCloud App

The app we developed can easily be used within the FeatureCloud platform. For this, a project coordinator creates a project, selects the app, and invites collaborators. Each participant installs FeatureCloud and joins the project. The app expects 2 CSV files as input, one for the training data and another for the test data. A config file can be used to define hyperparameters and other descriptors, such as the time and event label columns. After the federated computation has finished, each client receives the globally trained model as a pickle file, as well as a prediction file containing all predictions on the local test data set. The app can also be used in a FeatureCloud workflow, supporting various preprocessing methods, such as CV, normalization, feature selection, one-hot encoding, and subsequent evaluation of survival models using the c-index.

The requirements for running the survival SVM app are the same as for executing the FeatureCloud platform. It requires a stable internet connection to exchange the incentive model parameters with the central aggregator and to run the app on the website. Docker needs to be installed on a Mac, Linux, or Windows computer with the corresponding requirements for running Docker [39]. Moreover, enough memory should be available to process the data set. This depends mainly on the data set size, and not on the algorithm itself.

## Discussion

### Principal Findings

Our federated survival SVM has been demonstrated to offer a highly viable alternative to centralized data collection in a time-to-event analysis. It achieves comparable levels of accuracy without compromising the privacy of highly sensitive patient data. This makes it a compelling solution for organizations seeking to safeguard sensitive data while still gaining the benefits of advanced analysis and the application of ML. Through its availability as a FeatureCloud app, the platform takes care of deployment and federated infrastructures, making it directly usable with little programming knowledge. The results of the real-world microbiome data set are promising and show that FL might be an accelerator in microbiome research and the analysis of time-to-event microbiome data sets. Using FL combined with additive secret sharing, our approach can be currently considered GDPR compliant and, therefore, practically usable in real clinical time-to-event studies [12].

### Comparison to Existing Work

Only a few federated survival analysis approaches were developed in recent years, such as the distributed Cox proportional hazards model WebDISCO or an approach for federated survival curves using multiparty HE [18,20]. In a recent study about privacy-aware multi-institutional time-to-event analysis, it was criticized that the existing work was mainly focusing on theoretical solutions, rather than practical [21]. Therefore, lack of usability was a huge issue that was addressed by the authors, who developed the platform “Partea” [21]. The platform supports the Kaplan-Meier estimator for survival curve estimation [40], Nelson-Aalen estimator for cumulative hazard ratios [41], and Cox proportional hazards model for survival regression [42]. Compared with “Partea,” FeatureCloud does not only address the execution of FL algorithms, but also development. The FeatureCloud developer application programming interface for implementing FL algorithms that can be executed through FeatureCloud and published in the App Store is a huge advantage in terms of development speed and also accessibility for the potential user group.

To our knowledge, the survival SVM FeatureCloud app is one of the first time-to-event analysis ML models implemented as a FL algorithm. This makes the accuracy (or c-index in our case) between the algorithms not directly comparable. However, similar to the existing solutions [20,21], our approach achieves almost identical results compared with the central algorithms.

Regarding runtime, univariate methods without iterations, such as Kaplan-Meier estimator, Nelson-Aalen estimator, or log-rank test are much more efficient in FL settings. However, these approaches cannot be used to analyze high dimensional data and multivariate settings. The efficiency of our approach is comparable to the iteratively trained Cox proportional hazard model, which is trained iteratively and requires communication and aggregation for every parameter update step.

### Limitations

Our current approach does not support the more efficient ranking objective, as federated ranking is not trivial to implement. Instead, it is based on *scikit-survival*’s regression objective. Moreover, it solely supports the linear SVM and does not support the kernel SVM yet. Calculating a kernel matrix in a federated setting is not trivial, as it represents pairwise similarities (or distances) between the training data points. For supporting more complex, nonlinear relationships, this should be further investigated in the future. We still decided to implement and use a survival SVM in this work, as SVMs are very popular in health care and the only available time-to-event analysis ML model in *scikit-survival* that is not based on an ensemble approach. Ensemble models, such as random survival forests [43] or survival gradient boost, are both based on a set of survival trees. While ensemble models are also popular in time-to-event analysis, the federated aggregation of the local forests produces slightly worse results than centrally trained models in imbalanced scenarios [44]. A federated aggregation of each local tree, on the other hand, is computationally costly. The SVM in our implementation produces highly accurate results compared with central learning for model weights, c-index, and feature importance and can therefore lower the burden of applying FL in health care (eg, microbiome analysis), as the participants can be sure that the results are equal to the ones they would obtain in a central setting.

FeatureCloud currently only supports a simple additive secret-sharing scheme, increasing runtime for calculations with many clients and iterations. This could be solved in the future by using a more efficient secret-sharing scheme, such as Shamir secret sharing, that is currently not supported by FeatureCloud [45]. By using FeatureCloud as the execution platform, our approach does not solve the still existing open problems of FL, such as fairness, debugging, and communication efficiency (especially when using secret sharing) [46]. Furthermore, there are attacks on FL architectures that cannot be prevented through the existing methods, such as privacy inference from the global model, and model or data poisoning [47]. It is therefore recommended to use the algorithms and FeatureCloud platform only with trusted parties.

Another limitation that comes from the FeatureCloud platform is data standardization. Data formatting and standards need to be discussed and determined in advance by the participants of the federated analysis. However, FeatureCloud provides the possibility to include federated data preprocessing applications in the workflow. While this does not remove the need for external communication of data standards, such as included features and naming conventions, it makes it straightforward to guarantee the same format and preprocessing for the used data before the actual model training process. Possible applications include imputation, normalization, train or test splitting, and CV [48,49].

### Conclusions

In conclusion, we developed an open-source federated survival SVM that performs time-to-event analysis on geographically distributed data sets without sharing sensitive raw data. It is freely available in the FeatureCloud App Store. The trained models are almost identical compared with centrally trained survival SVMs. This extends the palette of existing federated time-to-event analysis approaches by another algorithm that can be applied to various problems.

## Appendix

Multimedia Appendix 1 State workflow of the survival support vector machine (SVM) FeatureCloud app and difference between coefficients.

Multimedia Appendix 2 C-indices of central, federated, and federated + secure aggregation analyses.

Multimedia Appendix 3 Coefficients of the trained survival support vector machines (SVMs).

Multimedia Appendix 4 Correlation of Shapley additive explanations (SHAP) values between central, federated, and federated + secure aggregation model.

Multimedia Appendix 5 Shapley additive explanations (SHAP) beeswarm plots for the different models.

Multimedia Appendix 6 Runtime of the federated survival support vector machine (SVM) training with 1, 3, and 5 clients.

Multimedia Appendix 7 Runtime of the federated survival support vector machine (SVM) with 1, 3, and 5 clients of a large sample size synthetic data set.

## Data Availability

The data sets generated and analyzed during this study are available in the GitHub repository [50]. The code for the implementation of the federated survival SVM is available in the GitHub repository [51]. The microbiome data set is not publicly available due to privacy regulations but is available from the corresponding author on reasonable request.

## Abbreviations

BRCA: breast cancer data set
CV: cross-validation
DP: differential privacy
FL: federated learning
GBSG2: German Breast Cancer Study Group 2 data set
GDPR: General Data Protection Regulation
HE: homomorphic encryption
ML: machine learning
MSP: Metagenomic Species Pangenome
PET: privacy-enhancing technique
RSA: Rivest–Shamir–Adleman
SHAP: Shapley additive explanations
SMPC: secure multiparty computation
SVM: support vector machine
WHAS500: Worcester Heart Attack Study data set
