# Supplementary material for: Privacy-Preserving Federated Survival Support Vector Machines for Cross-Institutional Time-To-Event Analysis: Algorithm Development and Validation
Source: JMIR AI. 2024 Mar 29;3:e47652. doi: 10.2196/47652 (PMC11041494; doi:10.2196/47652)
Supplement: Multimedia Appendix 1 [file ai_v3i1e47652_app1.docx]

## Multimedia Appendix 1

## Figure S1: State workflow of the survival SVM FeatureCloud app


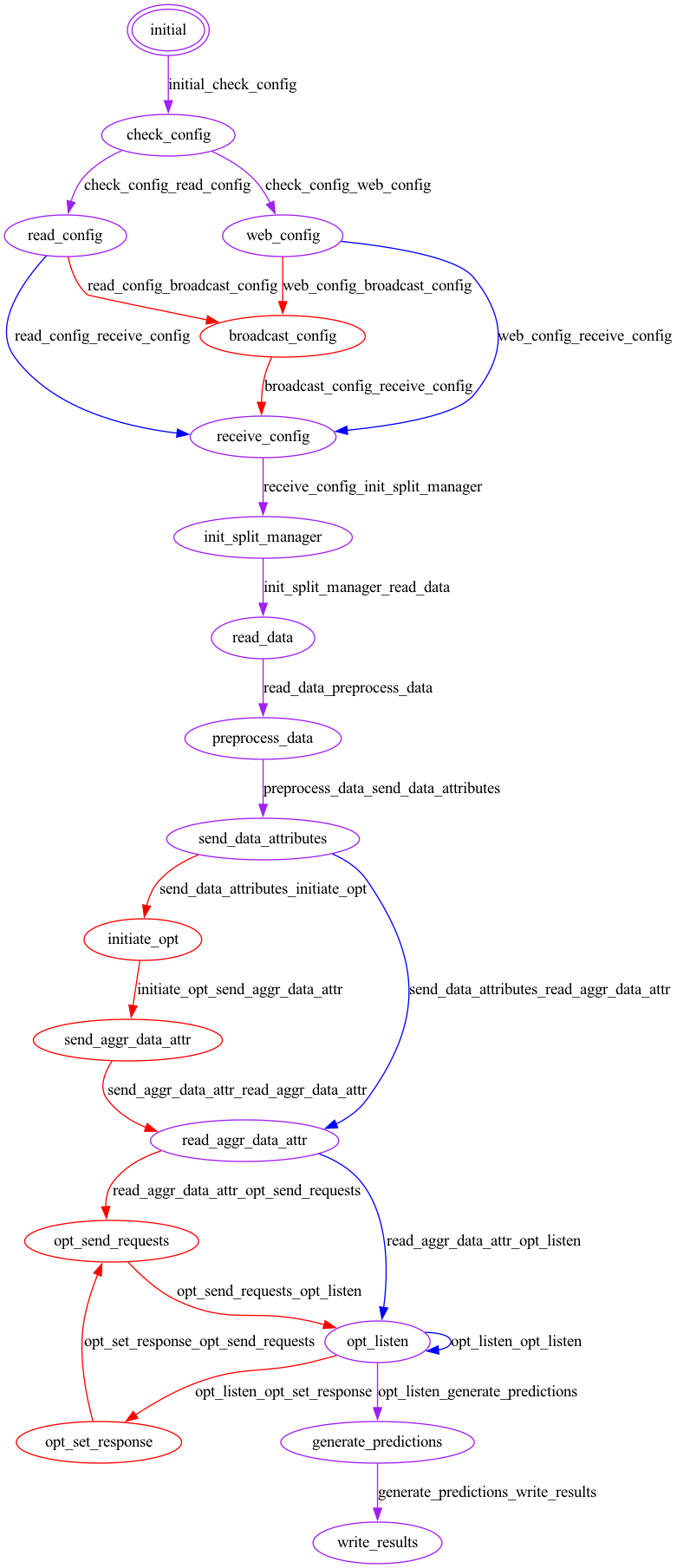


***Figure S1: States of the FeatureCloud app.*** *The states of the participants are shown in blue, states of the coordinator in red. Purple signals states that can be reached by participants and coordinator.*

## Figure S2: Difference between coefficients
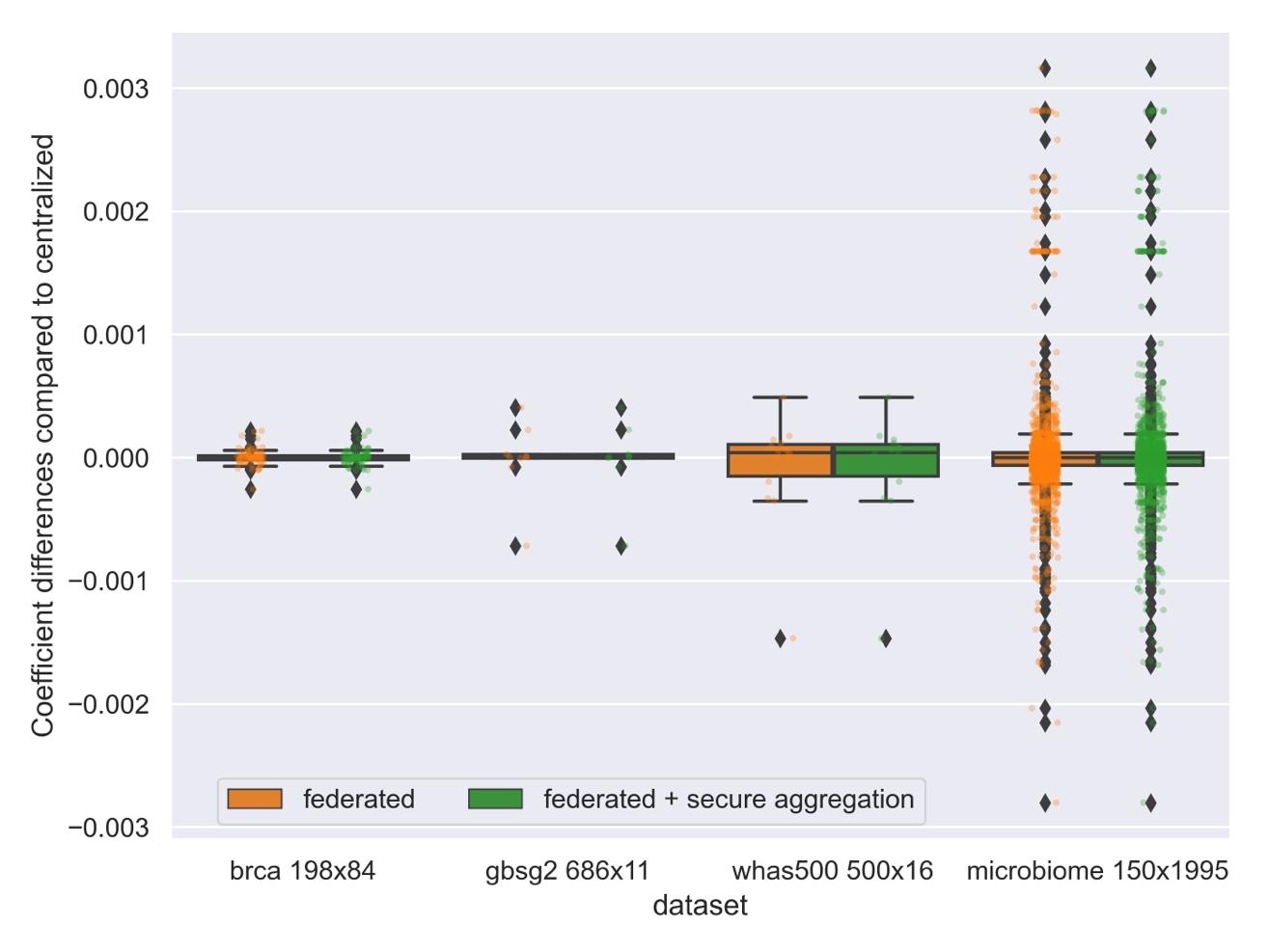


***Figure S2: Model weight differences for each dataset.*** *Each boxplot shows the differences in coefficients between our federated survival SVM and the centralized scikit-learn model for the four datasets. All upper percentiles show a lower difference than 0.0005. Some outliers occur, especially for the very high dimensional microbiome dataset with a large number of features and, therefore, model parameters; However, the largest difference between two coefficients is still below 0.0035.*
