# Supplementary figures and images for: Privacy-Preserving Federated Survival Support Vector Machines for Cross-Institutional Time-To-Event Analysis: Algorithm Development and Validation
Source: JMIR AI. 2024 Mar 29;3:e47652. doi: 10.2196/47652 (PMC11041494; doi:10.2196/47652)

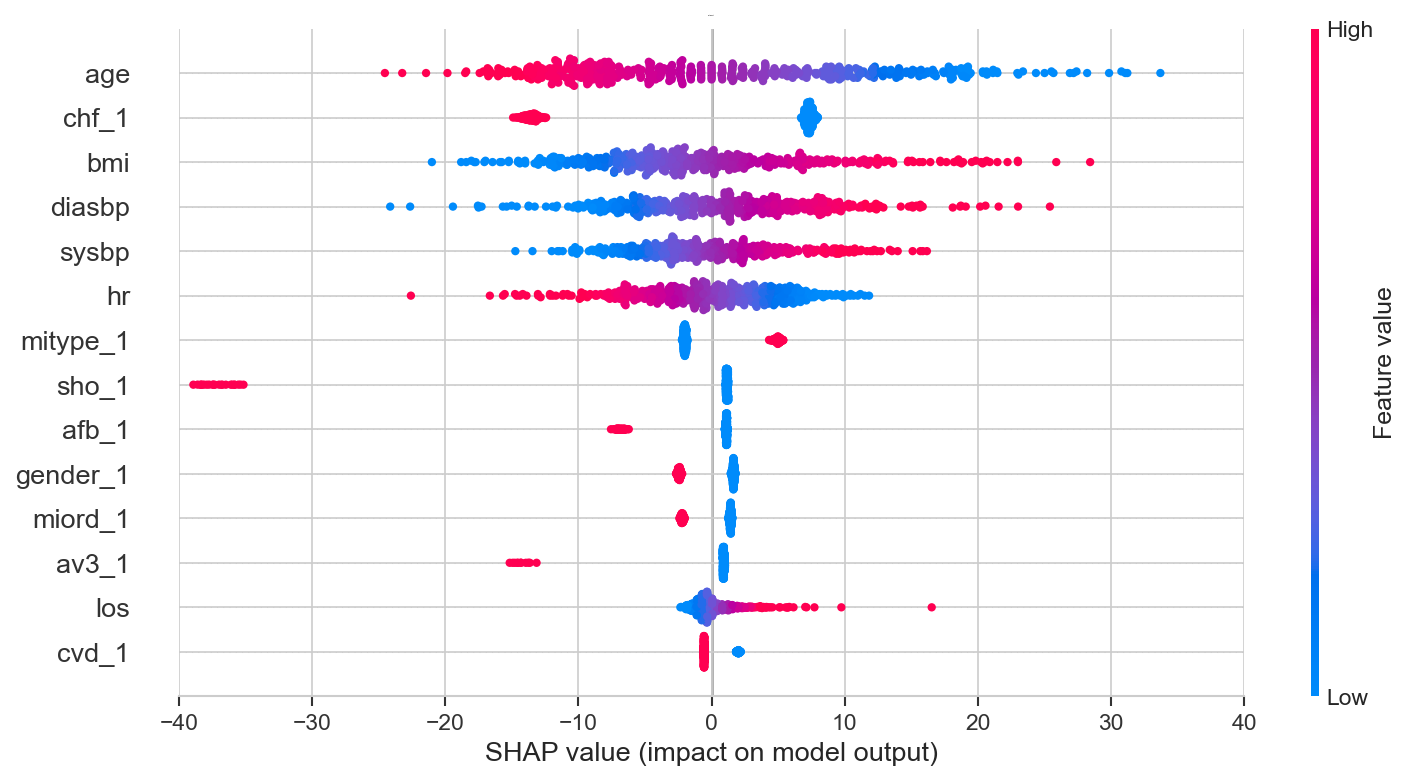

Supplement: Multimedia Appendix 5 [file ai_v3i1e47652_app5.zip › Publish/whas500/federated-analysis_dot.png]

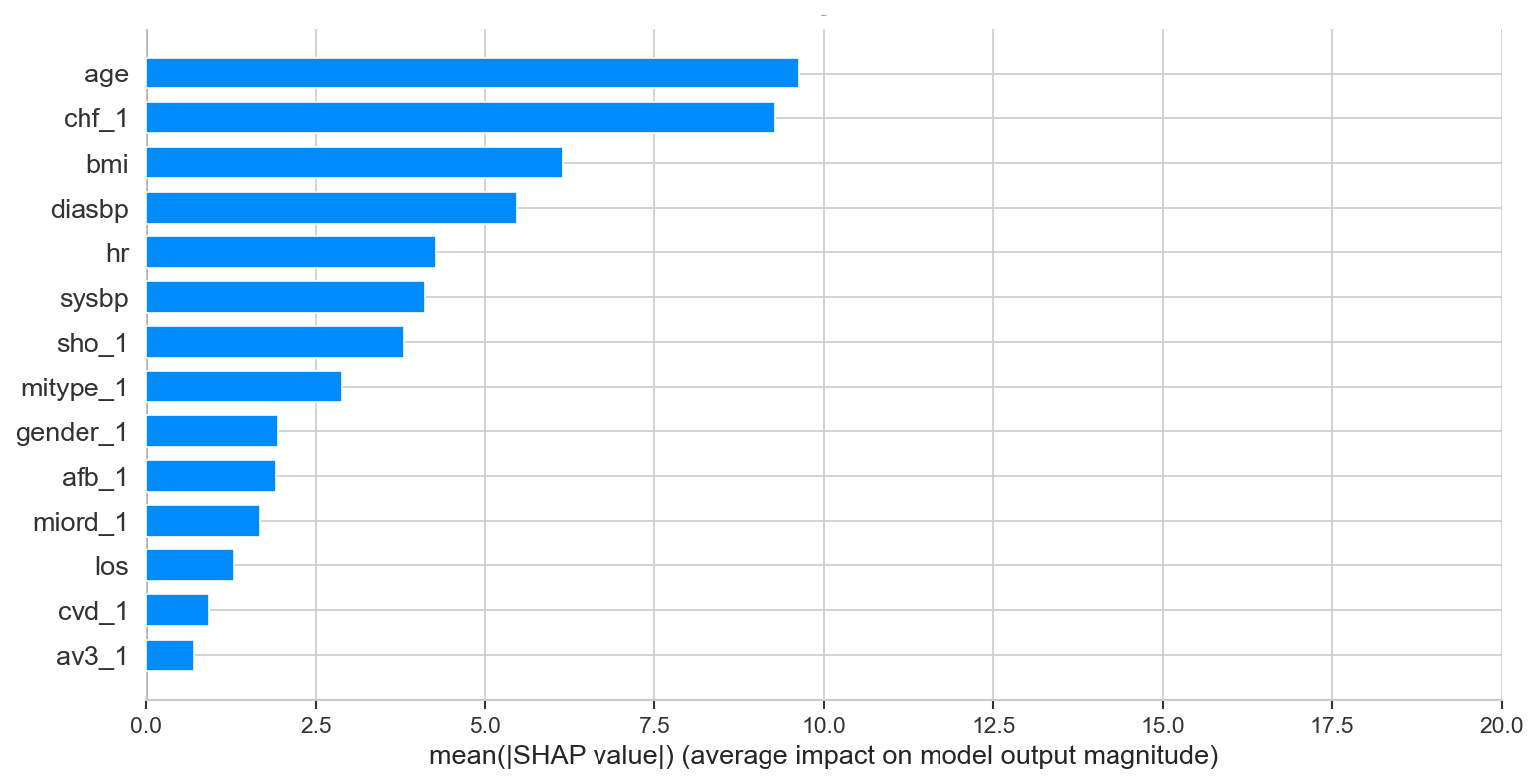

Supplement: Multimedia Appendix 5 [file ai_v3i1e47652_app5.zip › Publish/whas500/central_bar.png]

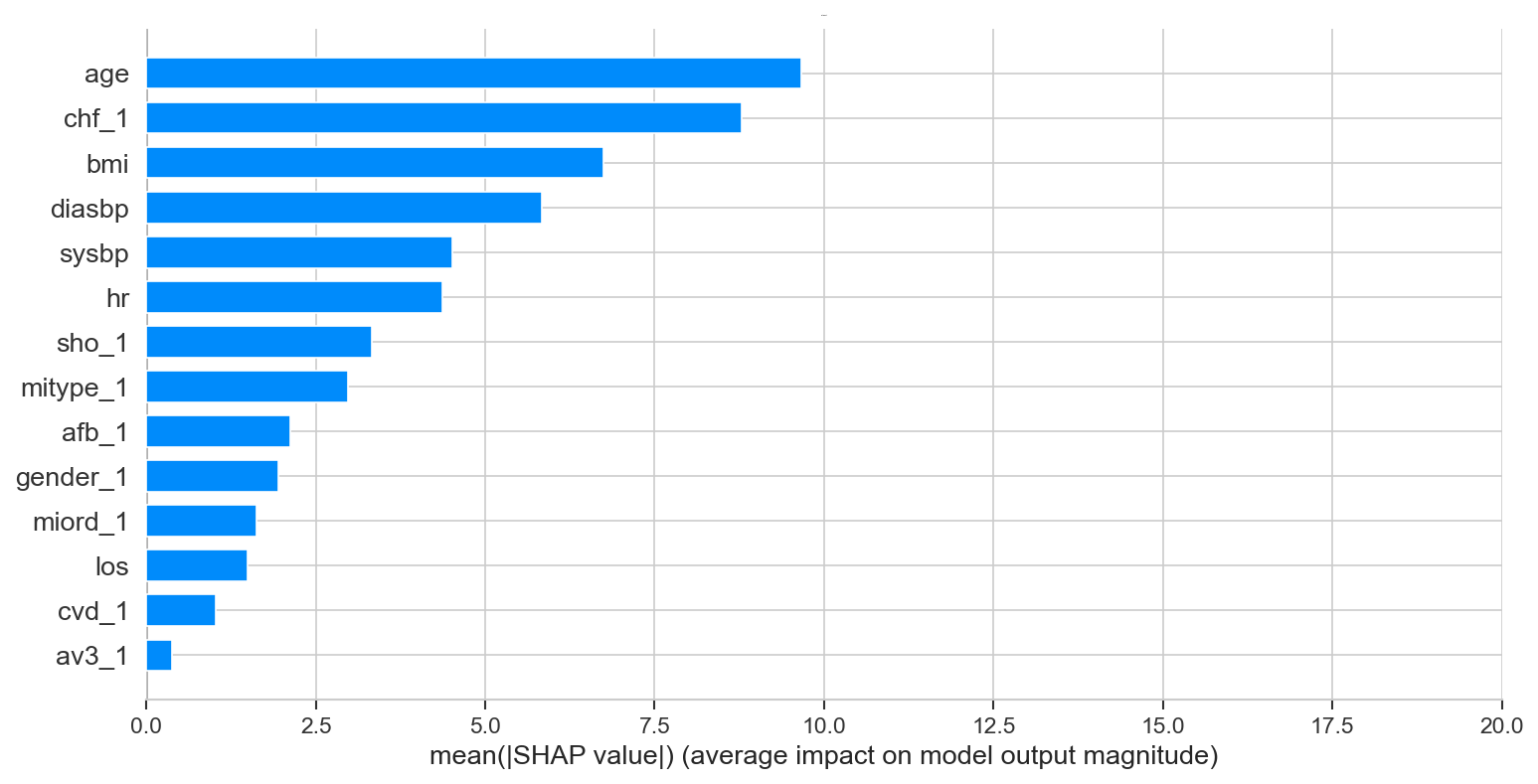

Supplement: Multimedia Appendix 5 [file ai_v3i1e47652_app5.zip › Publish/whas500/smpc-analysis_bar.png]

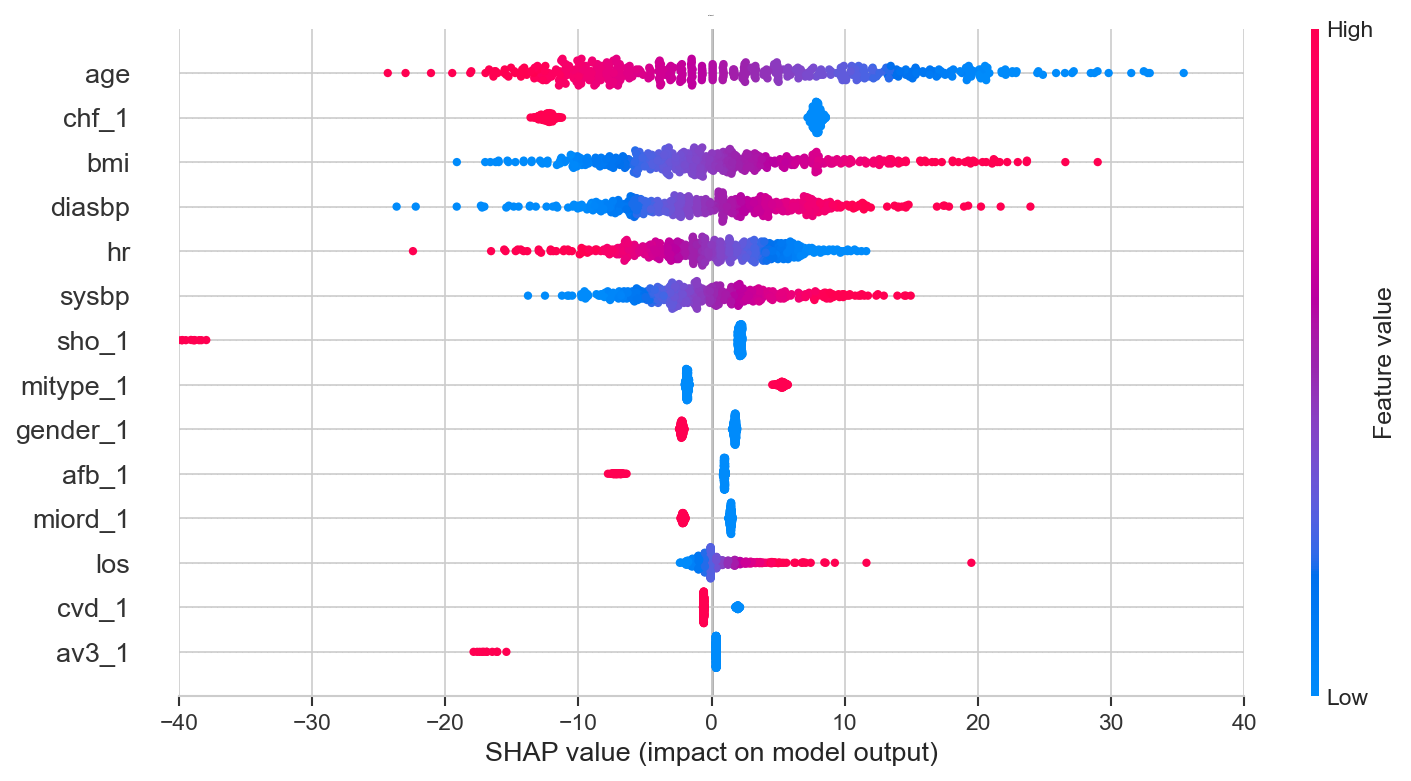

Supplement: Multimedia Appendix 5 [file ai_v3i1e47652_app5.zip › Publish/whas500/central_dot.png]

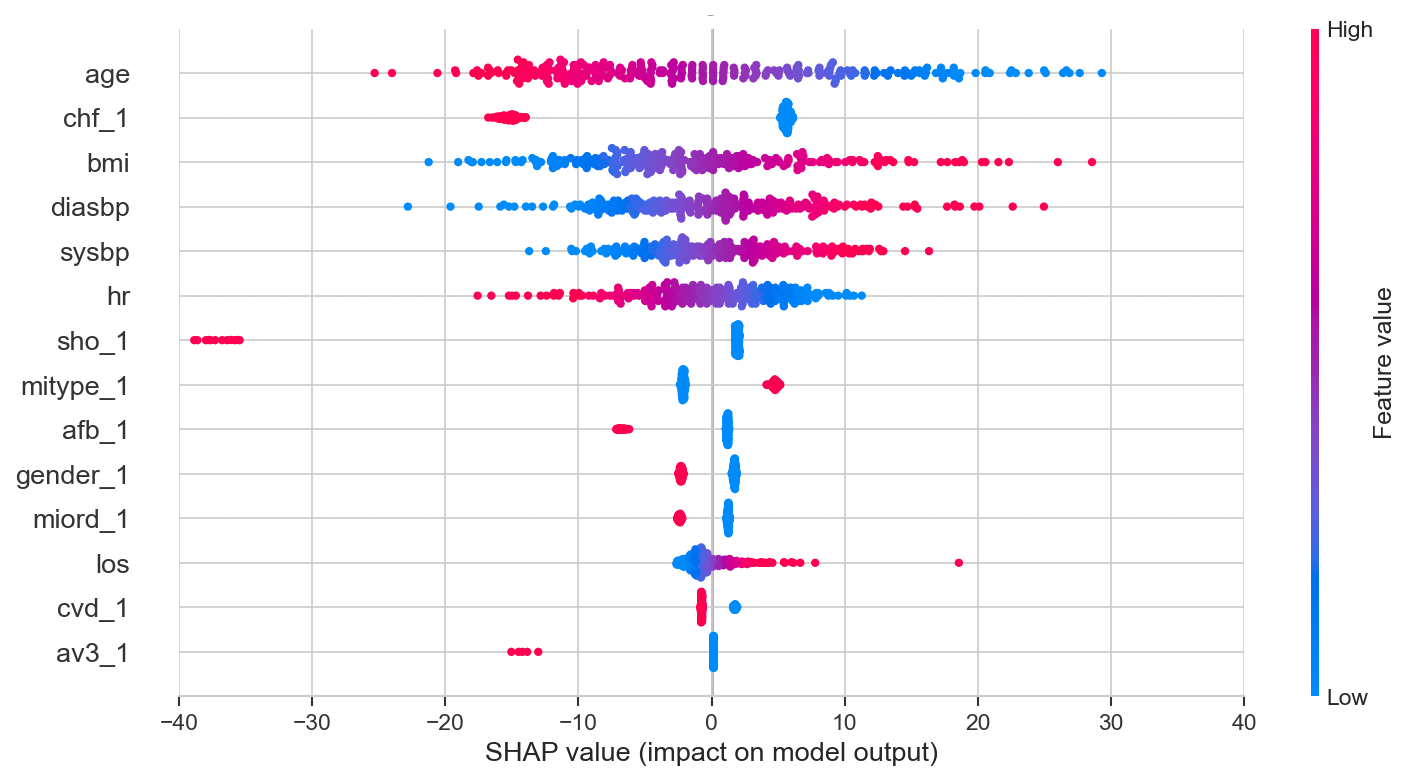

Supplement: Multimedia Appendix 5 [file ai_v3i1e47652_app5.zip › Publish/whas500/smpc-analysis_dot.png]

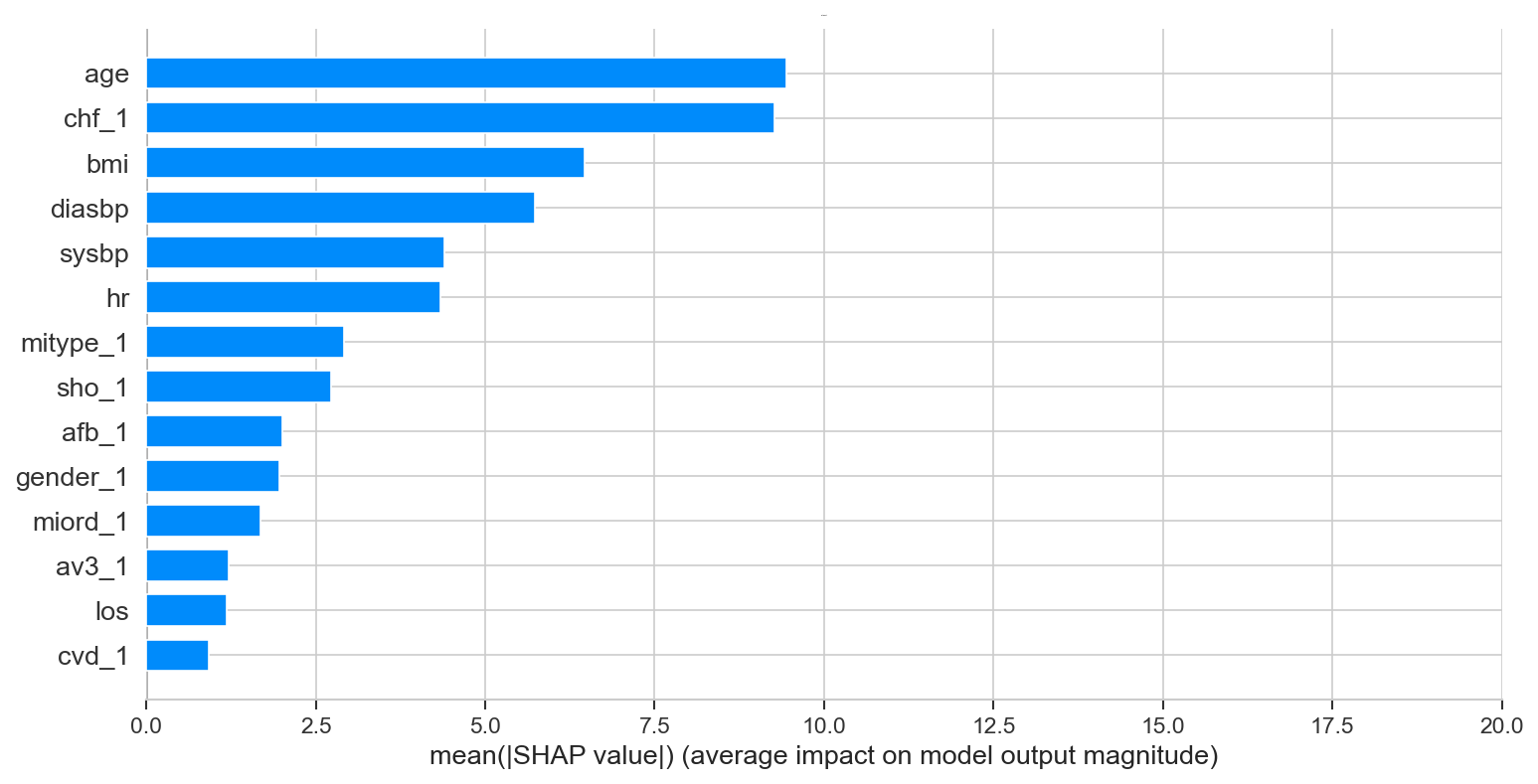

Supplement: Multimedia Appendix 5 [file ai_v3i1e47652_app5.zip › Publish/whas500/federated-analysis_bar.png]

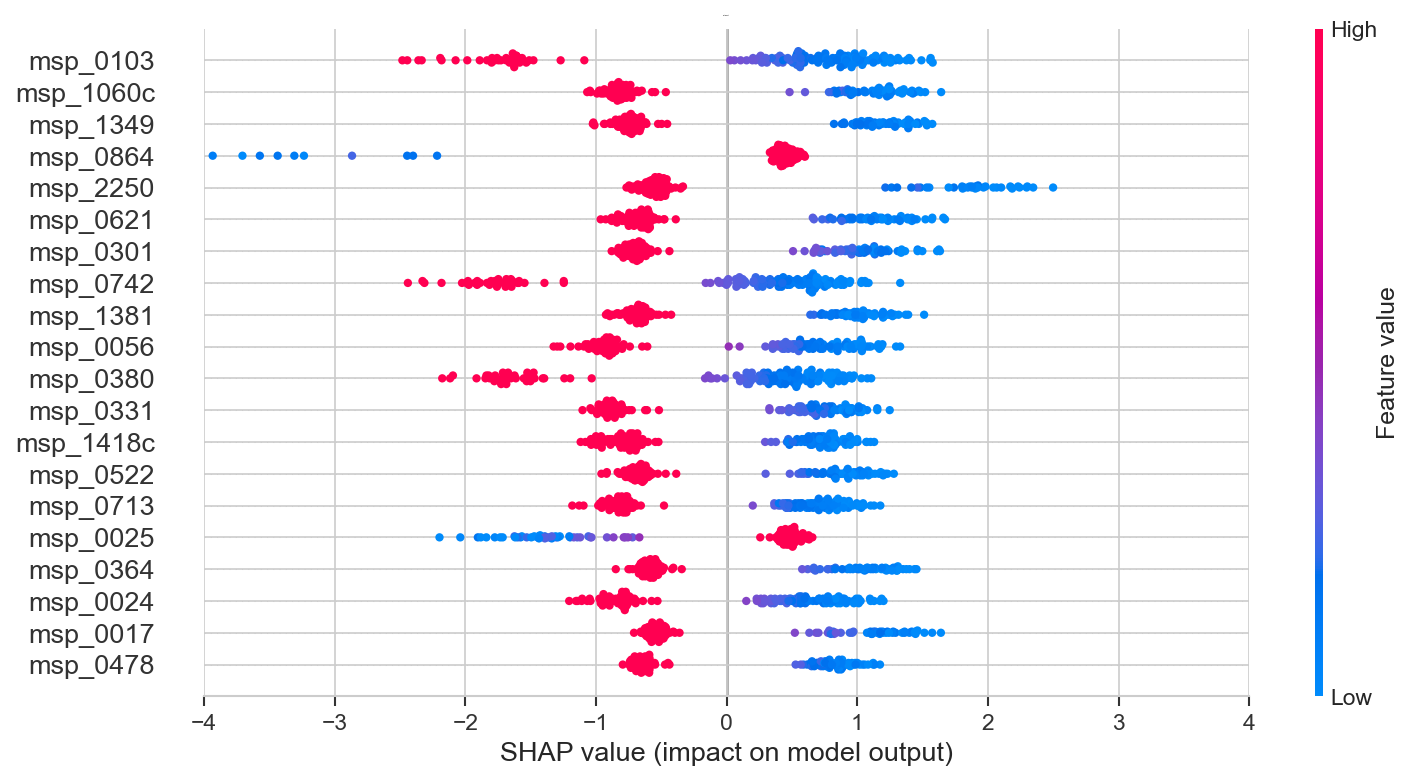

Supplement: Multimedia Appendix 5 [file ai_v3i1e47652_app5.zip › Publish/microbiome/federated-analysis_dot.png]

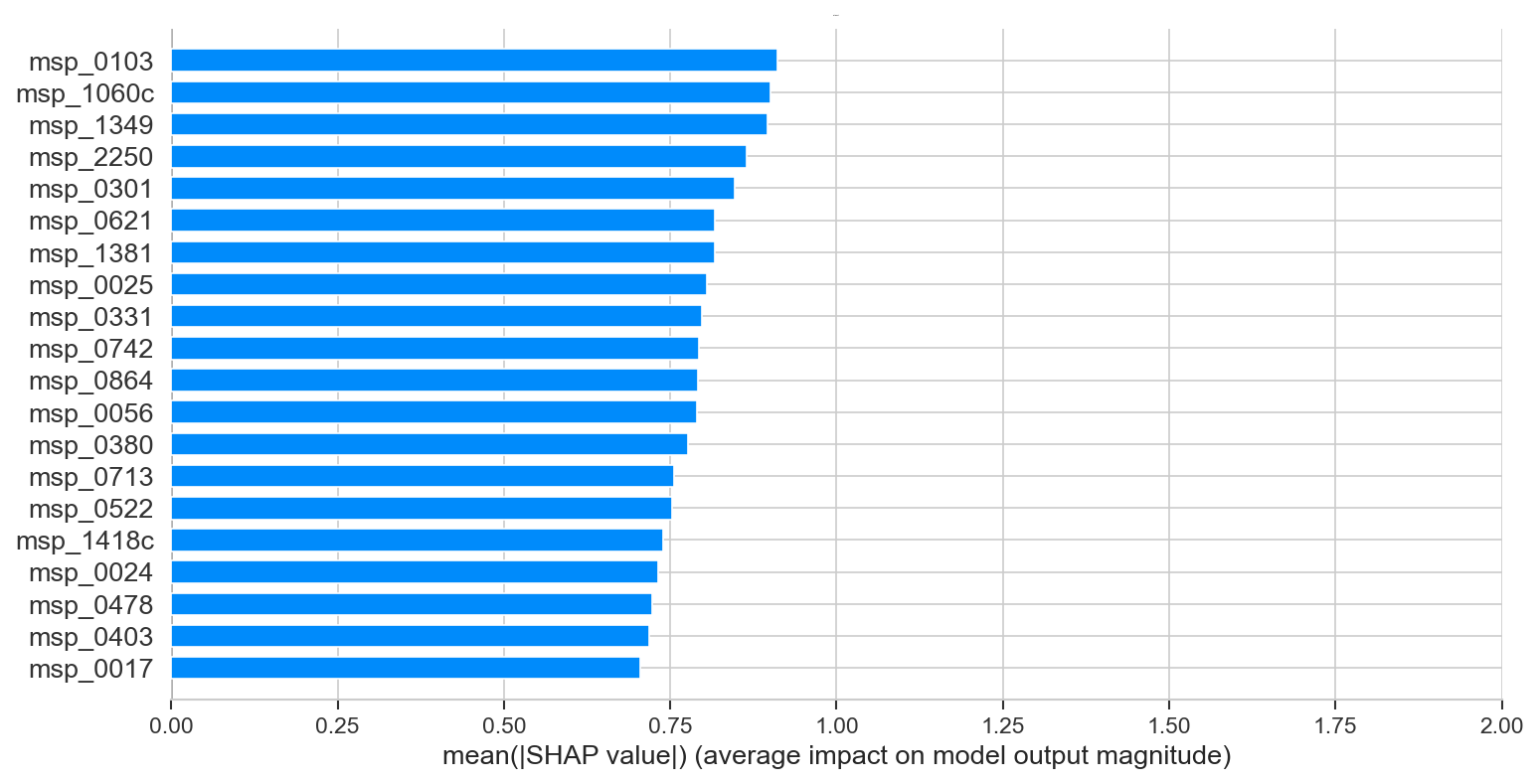

Supplement: Multimedia Appendix 5 [file ai_v3i1e47652_app5.zip › Publish/microbiome/central_bar.png]

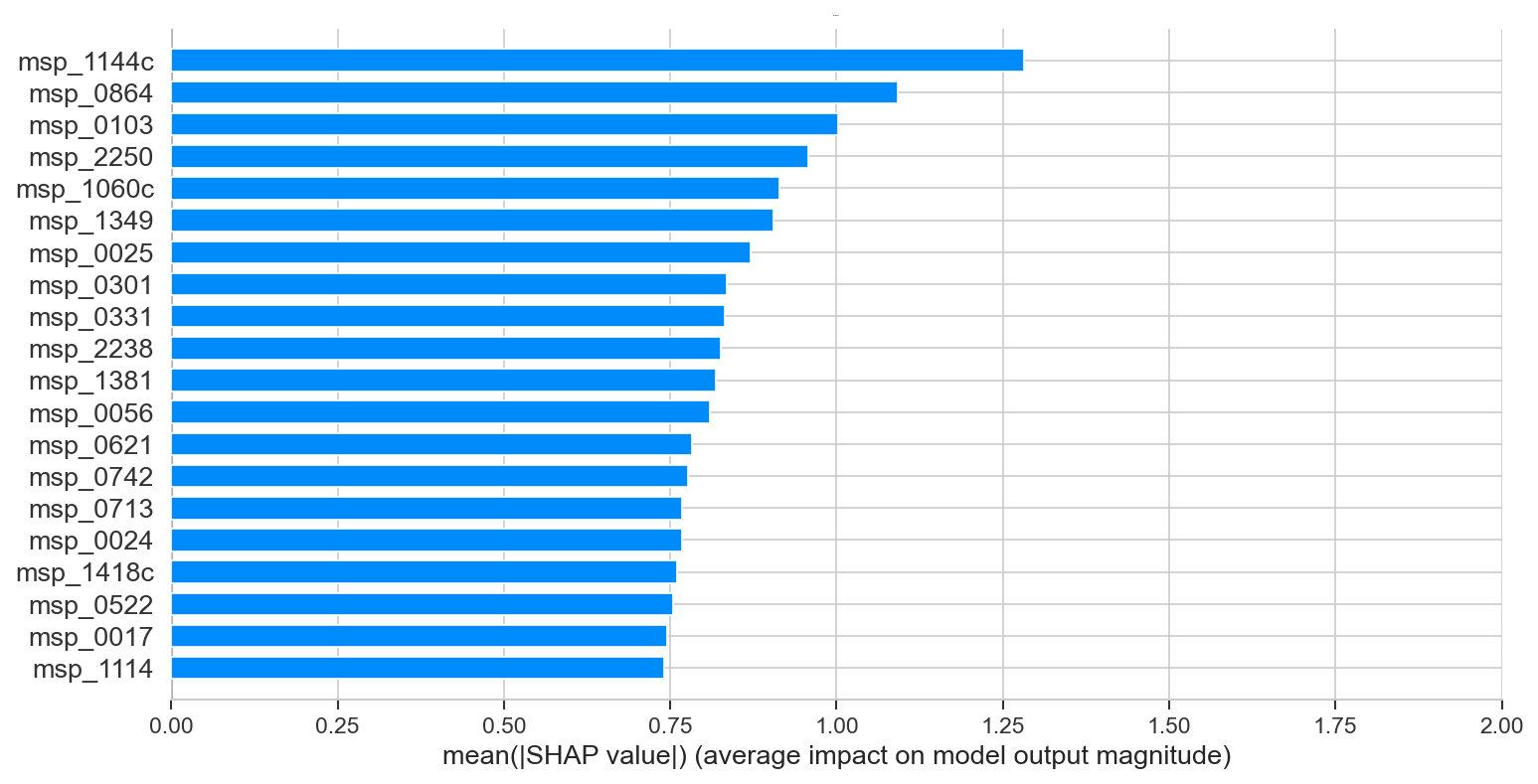

Supplement: Multimedia Appendix 5 [file ai_v3i1e47652_app5.zip › Publish/microbiome/smpc-analysis_bar.png]

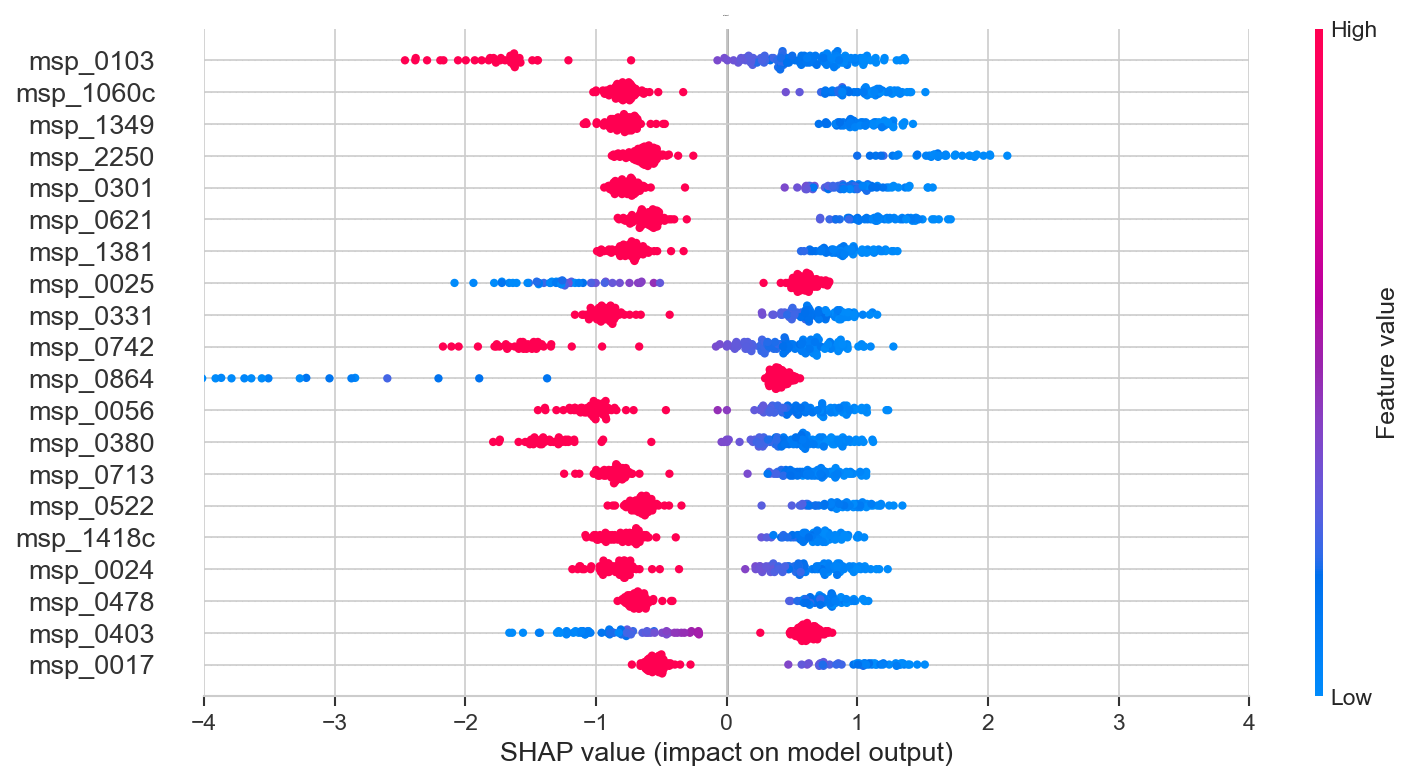

Supplement: Multimedia Appendix 5 [file ai_v3i1e47652_app5.zip › Publish/microbiome/central_dot.png]

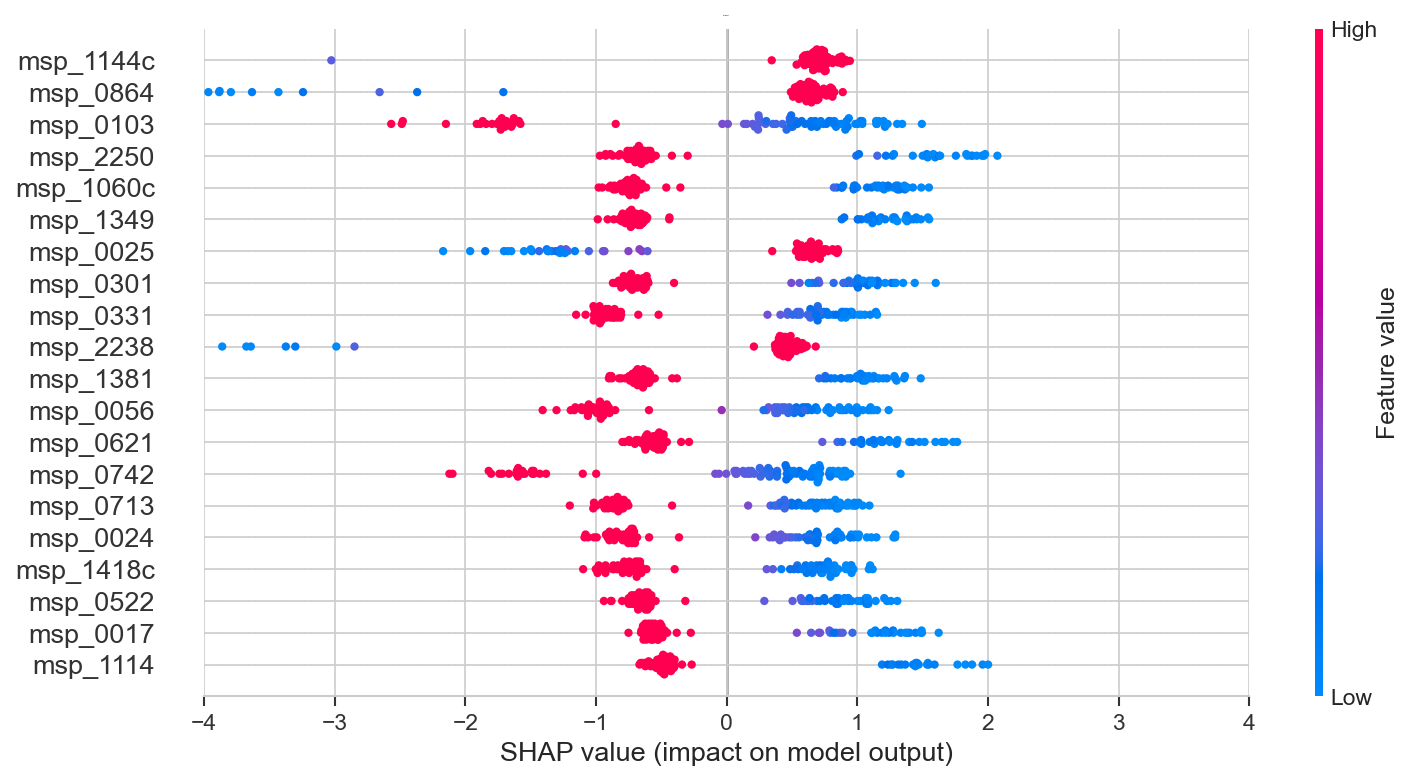

Supplement: Multimedia Appendix 5 [file ai_v3i1e47652_app5.zip › Publish/microbiome/smpc-analysis_dot.png]

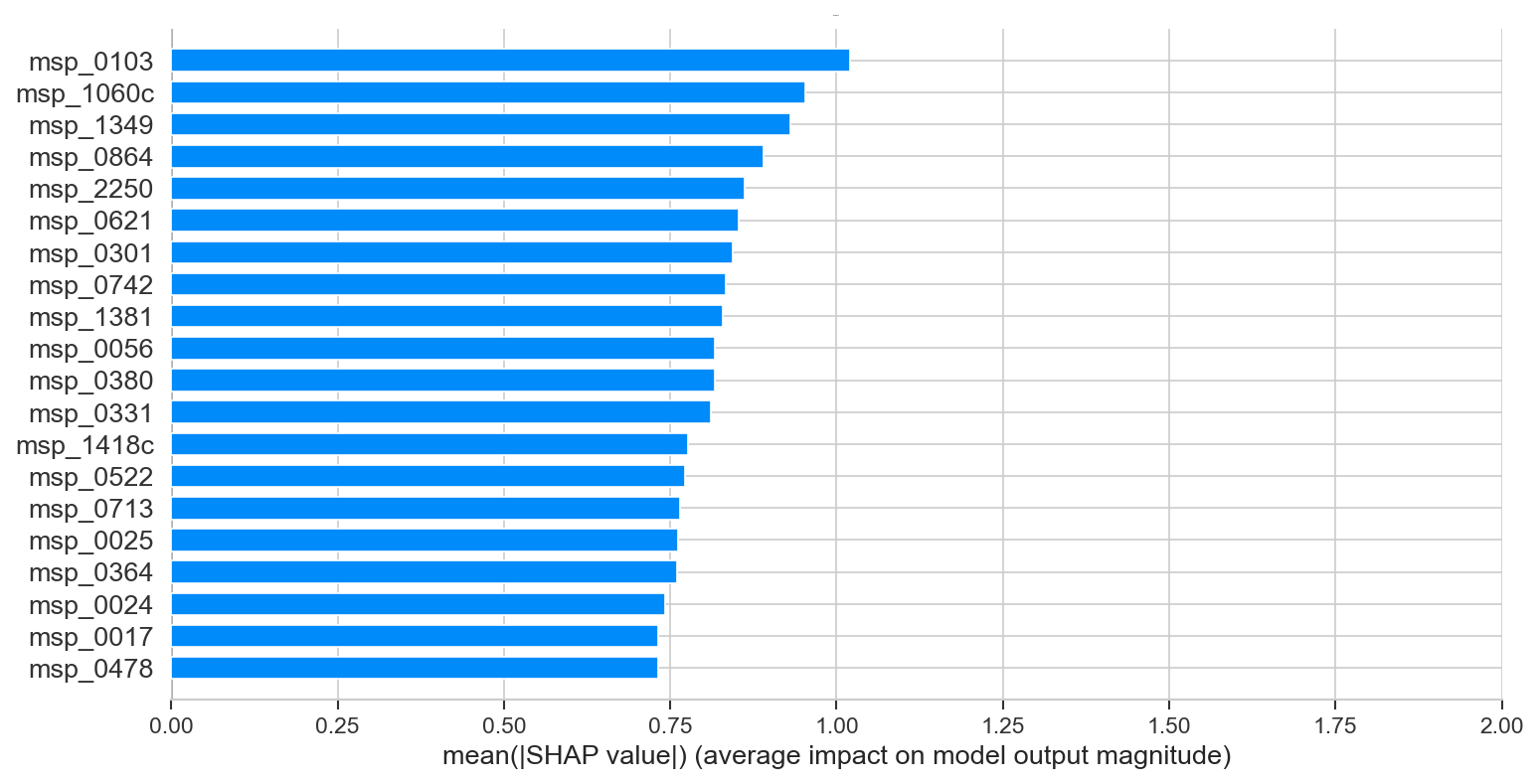

Supplement: Multimedia Appendix 5 [file ai_v3i1e47652_app5.zip › Publish/microbiome/federated-analysis_bar.png]

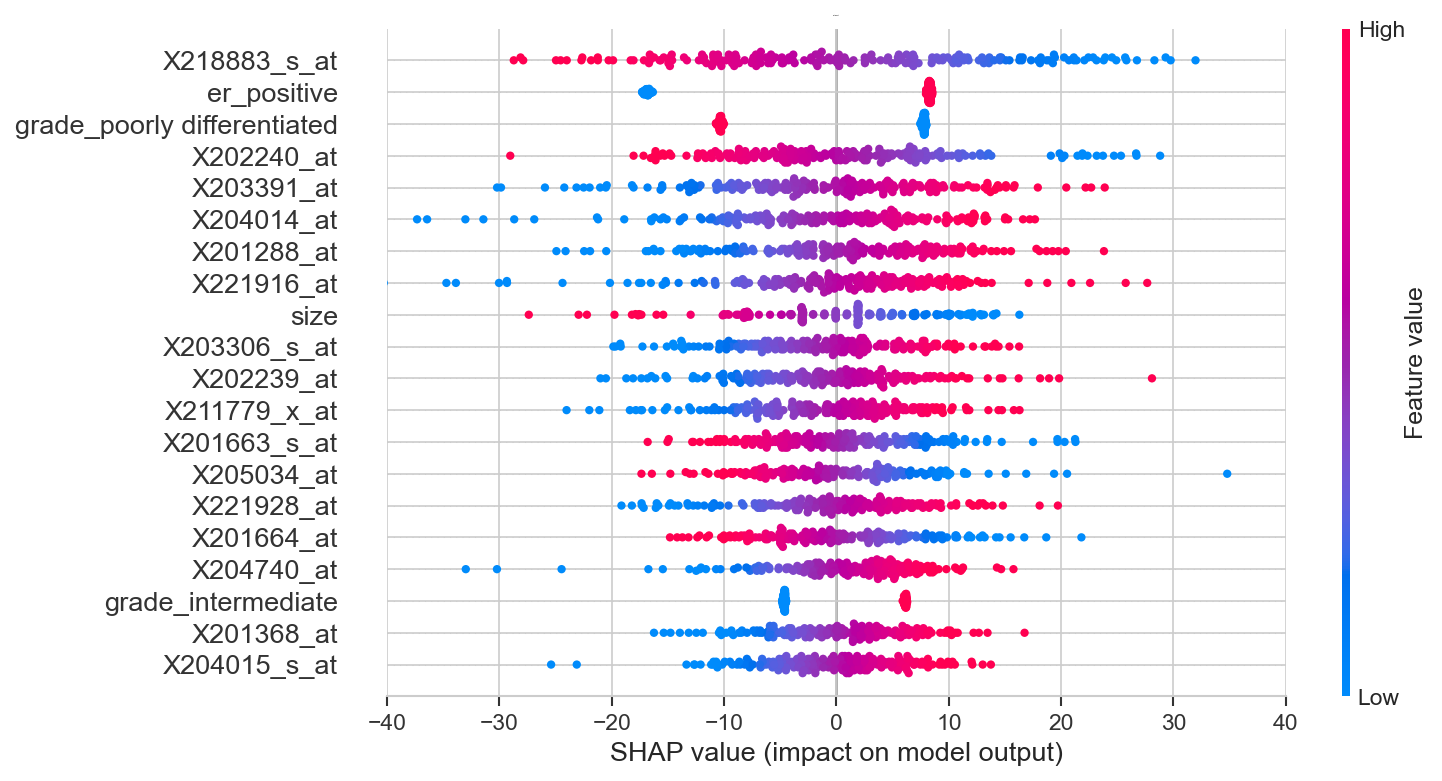

Supplement: Multimedia Appendix 5 [file ai_v3i1e47652_app5.zip › Publish/brca/federated-analysis_dot.png]

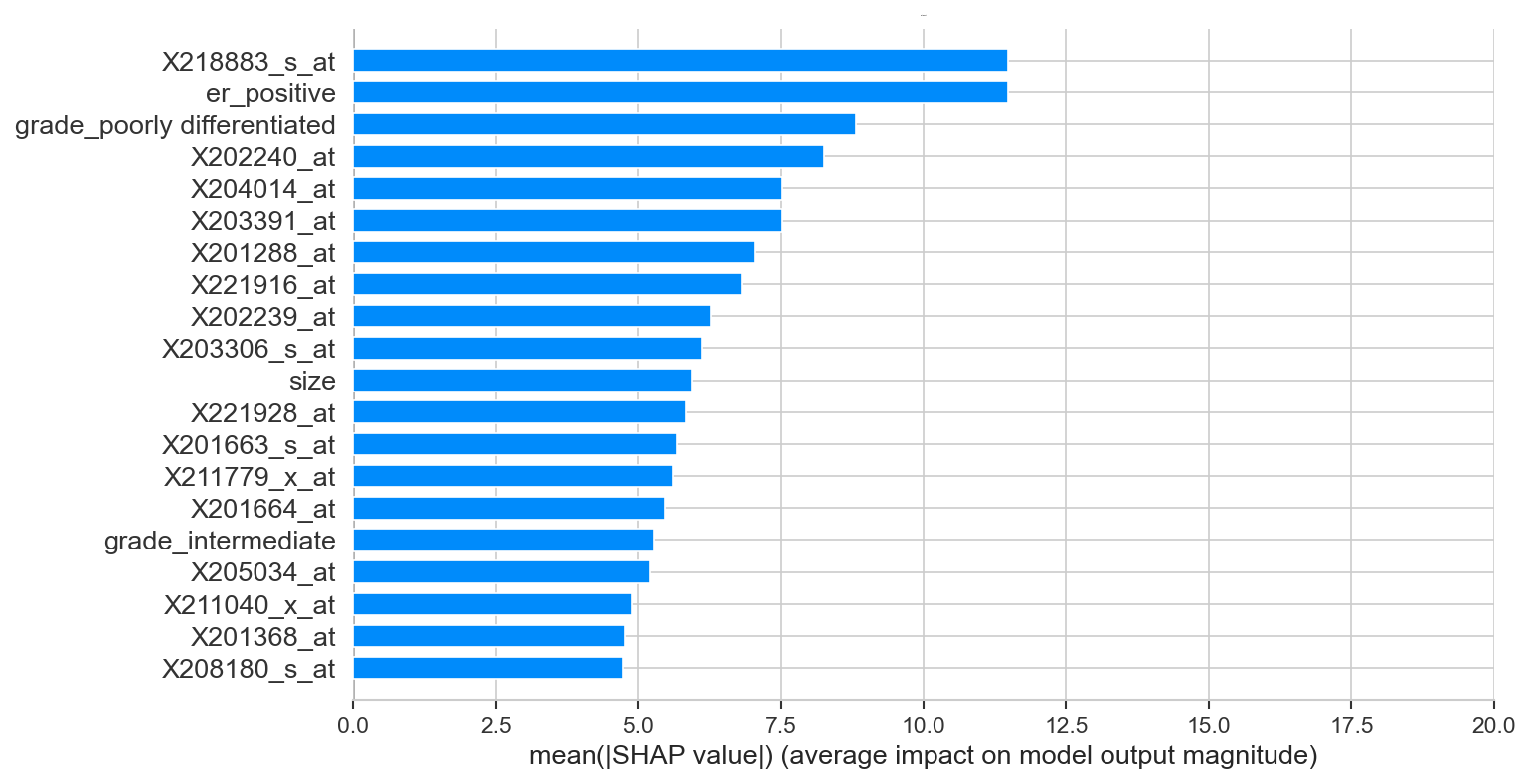

Supplement: Multimedia Appendix 5 [file ai_v3i1e47652_app5.zip › Publish/brca/central_bar.png]

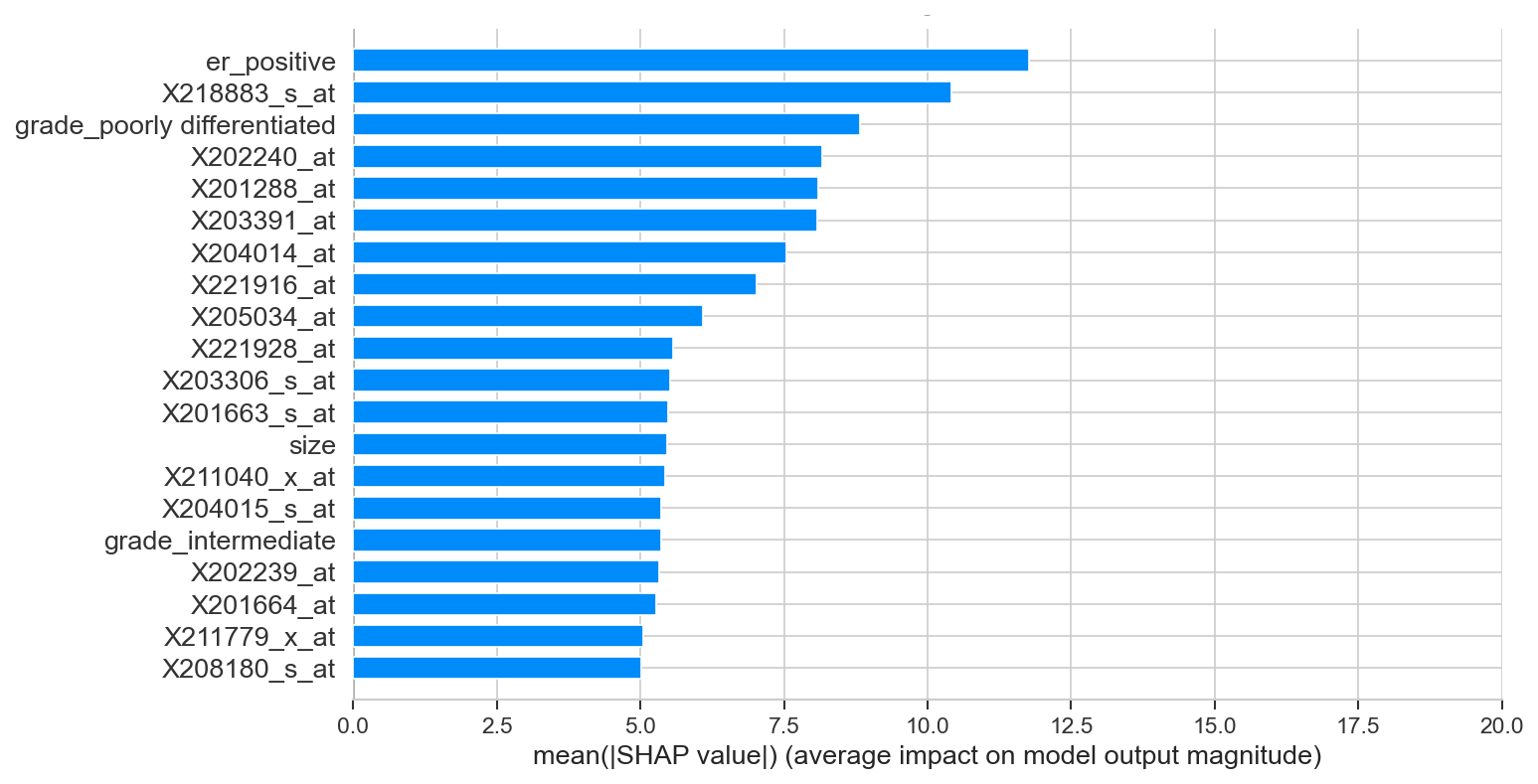

Supplement: Multimedia Appendix 5 [file ai_v3i1e47652_app5.zip › Publish/brca/smpc-analysis_bar.png]

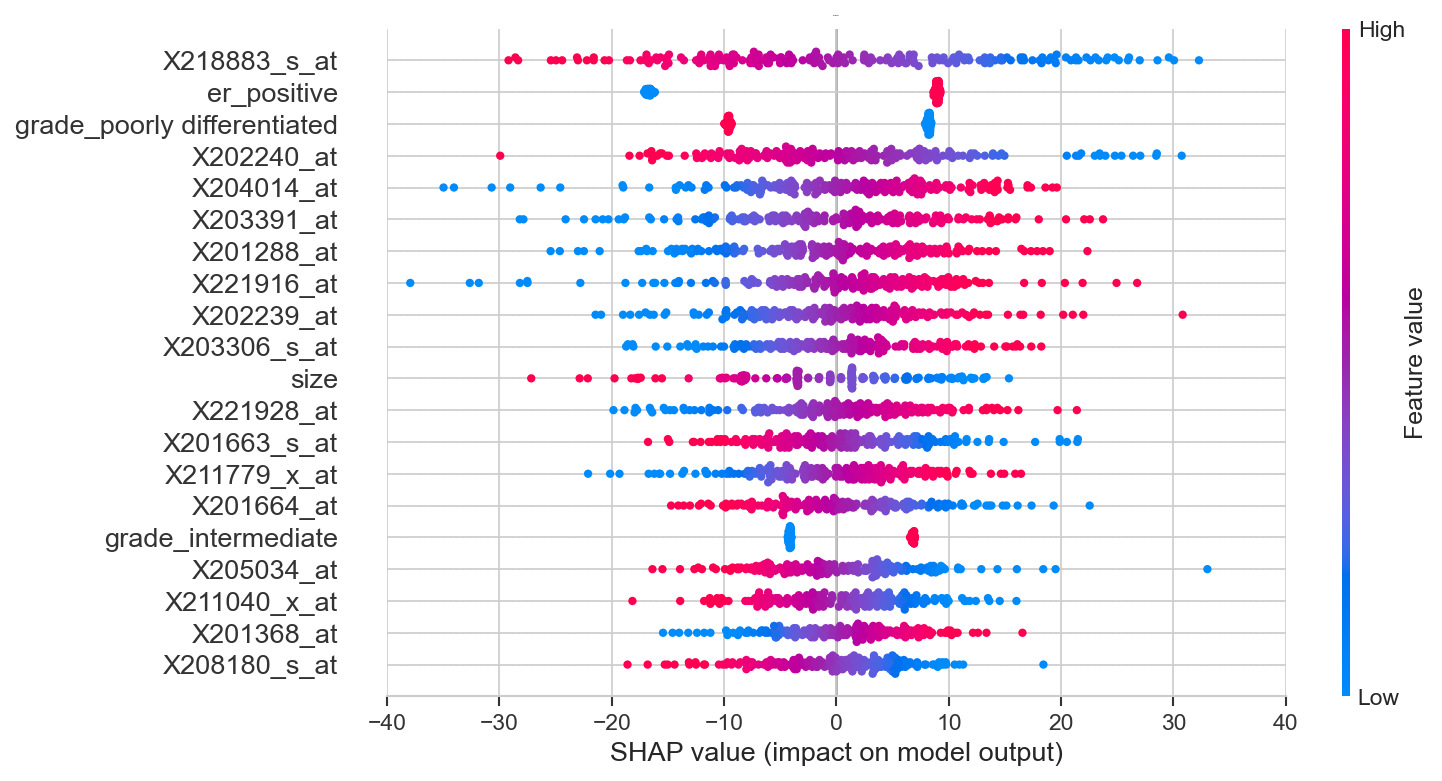

Supplement: Multimedia Appendix 5 [file ai_v3i1e47652_app5.zip › Publish/brca/central_dot.png]

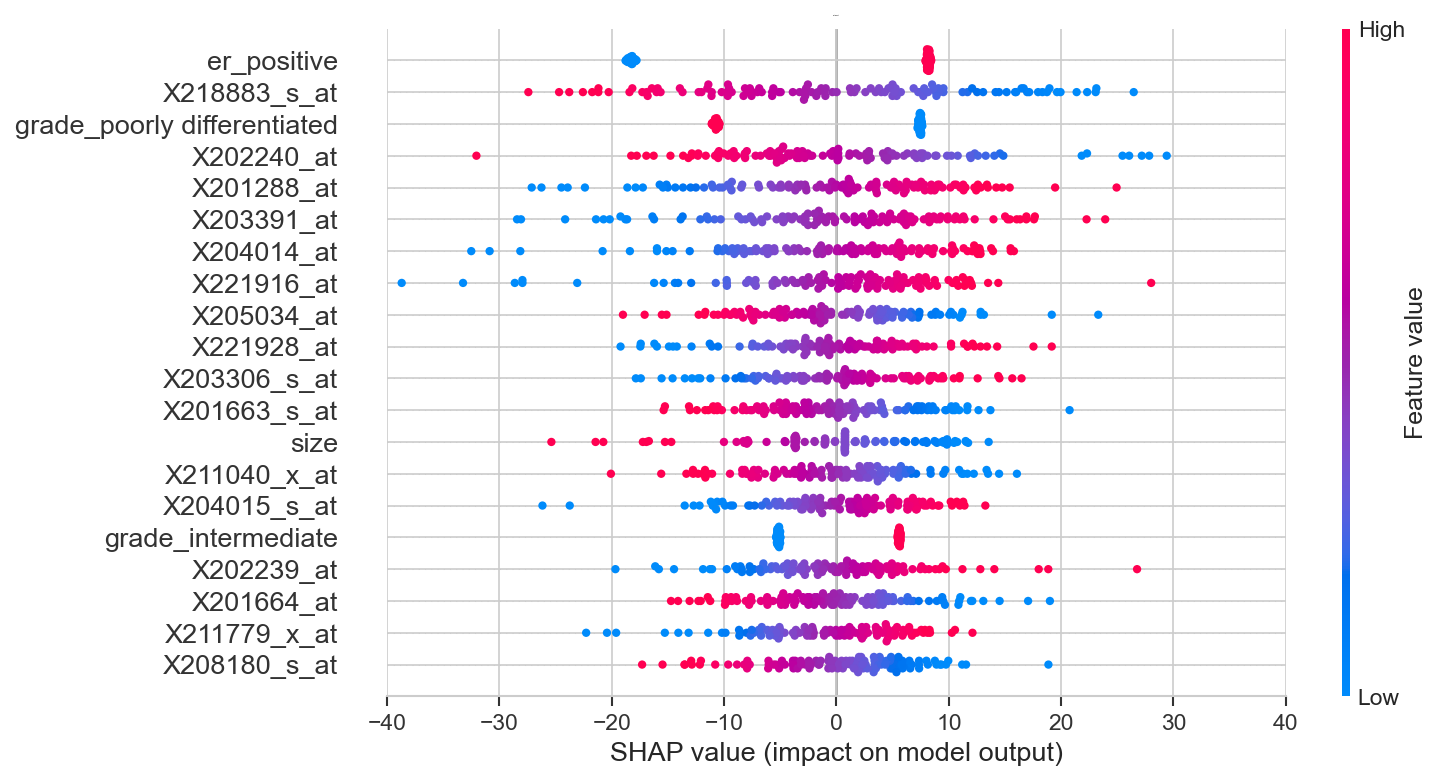

Supplement: Multimedia Appendix 5 [file ai_v3i1e47652_app5.zip › Publish/brca/smpc-analysis_dot.png]

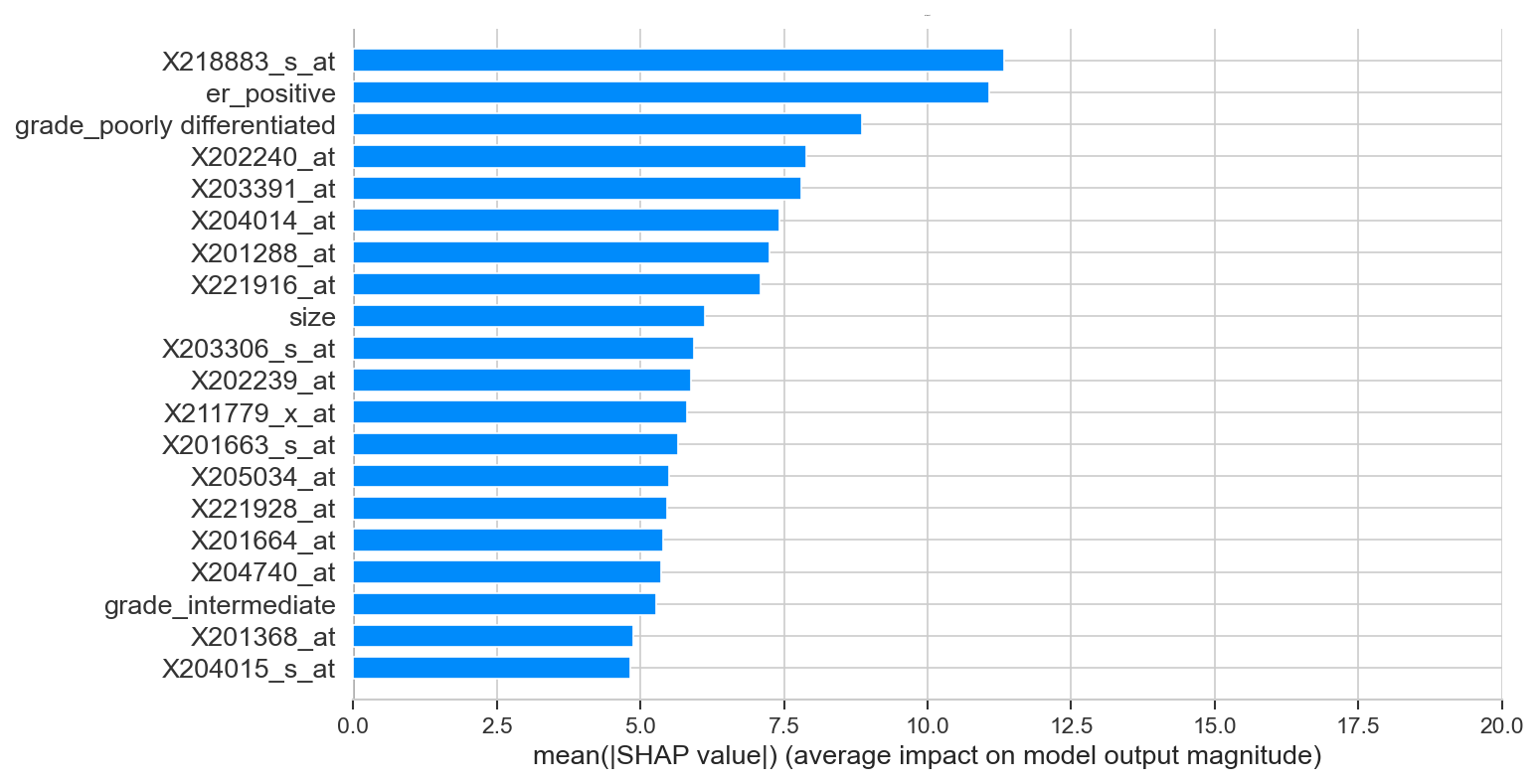

Supplement: Multimedia Appendix 5 [file ai_v3i1e47652_app5.zip › Publish/brca/federated-analysis_bar.png]

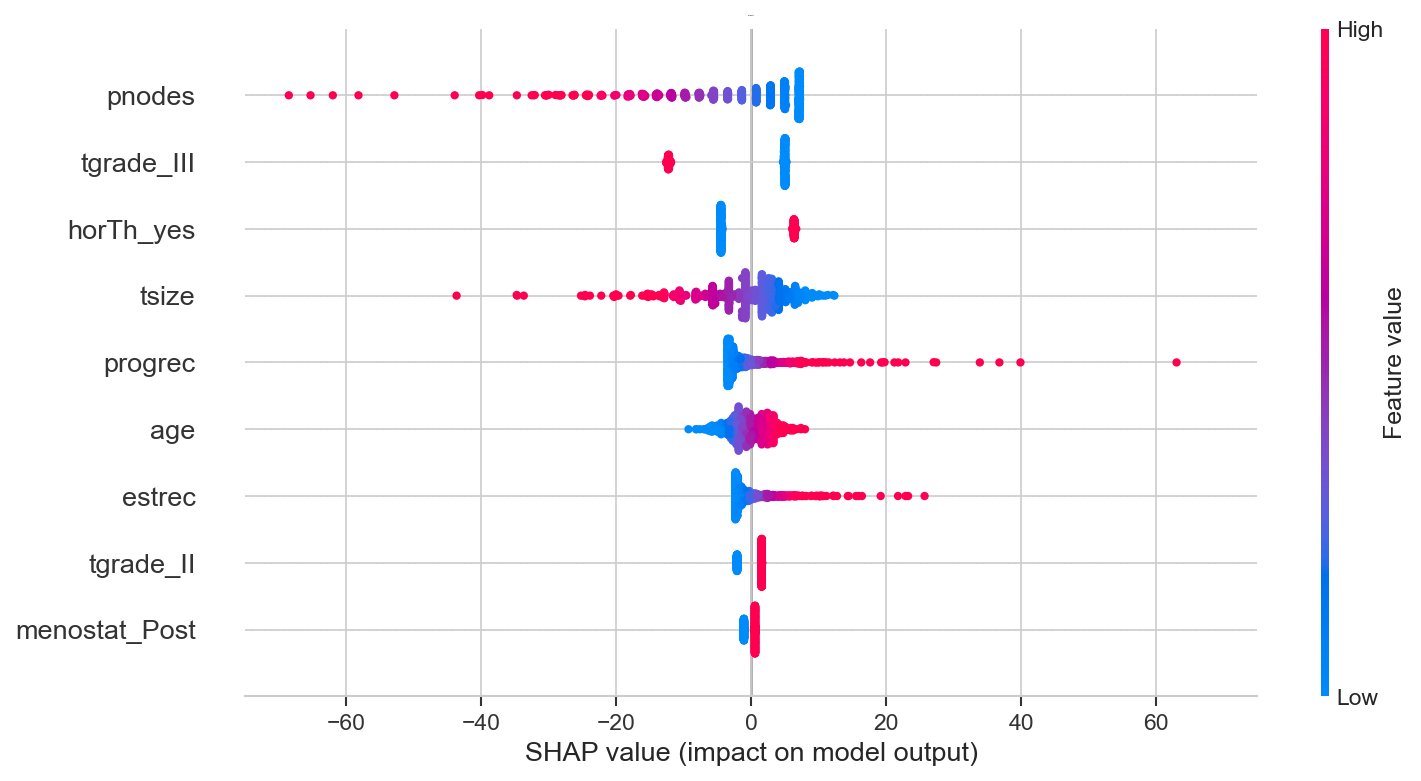

Supplement: Multimedia Appendix 5 [file ai_v3i1e47652_app5.zip › Publish/gbsg2/federated-analysis_dot.png]

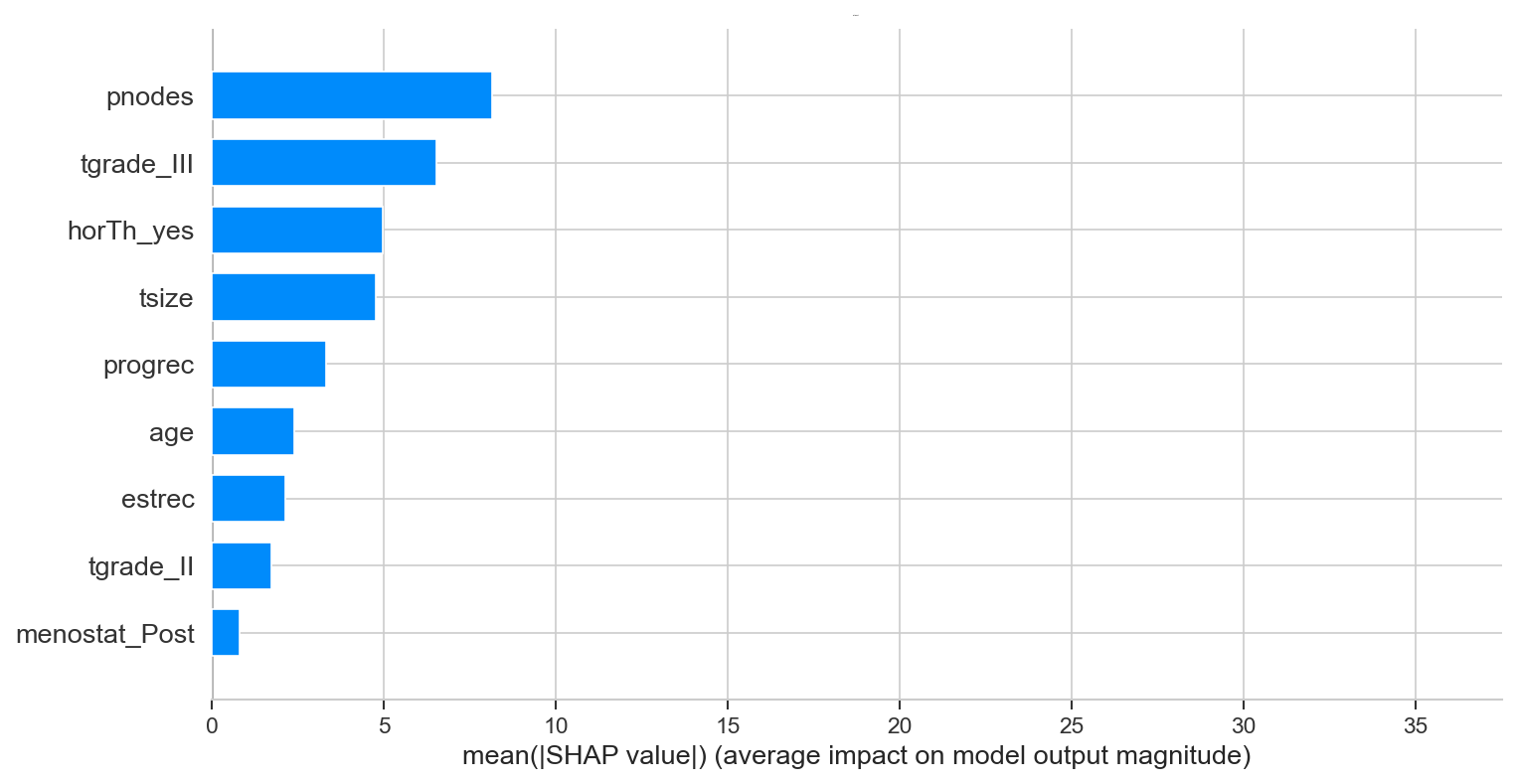

Supplement: Multimedia Appendix 5 [file ai_v3i1e47652_app5.zip › Publish/gbsg2/central_bar.png]

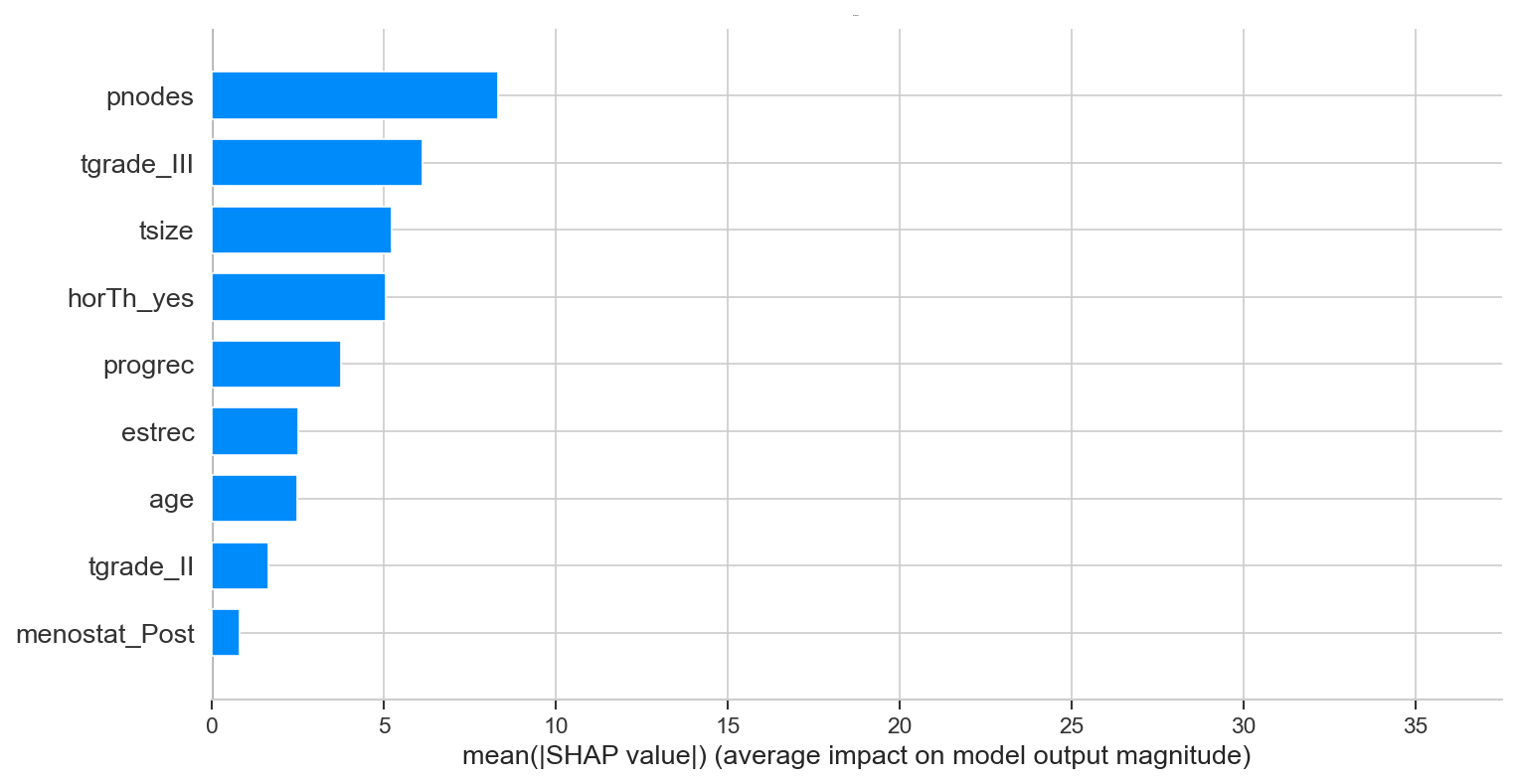

Supplement: Multimedia Appendix 5 [file ai_v3i1e47652_app5.zip › Publish/gbsg2/smpc-analysis_bar.png]

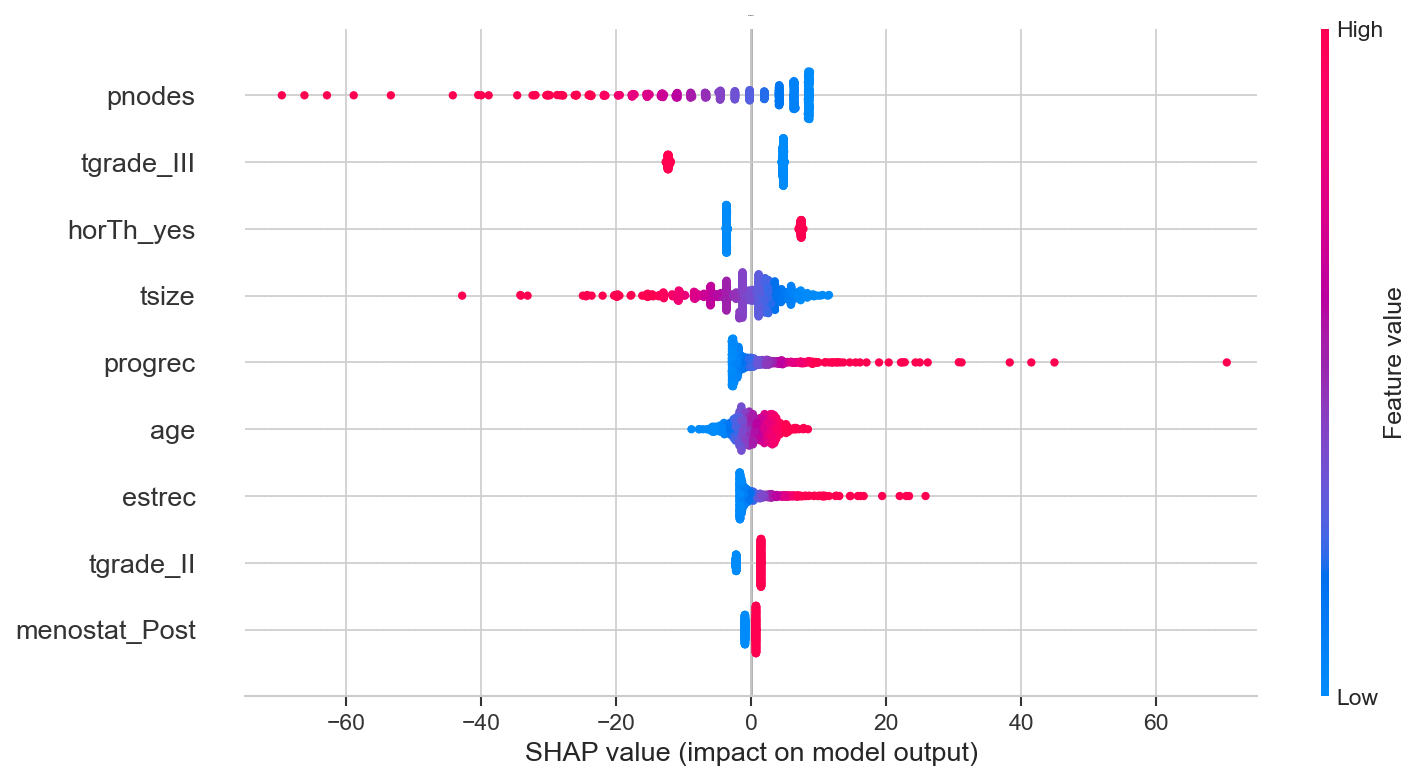

Supplement: Multimedia Appendix 5 [file ai_v3i1e47652_app5.zip › Publish/gbsg2/central_dot.png]

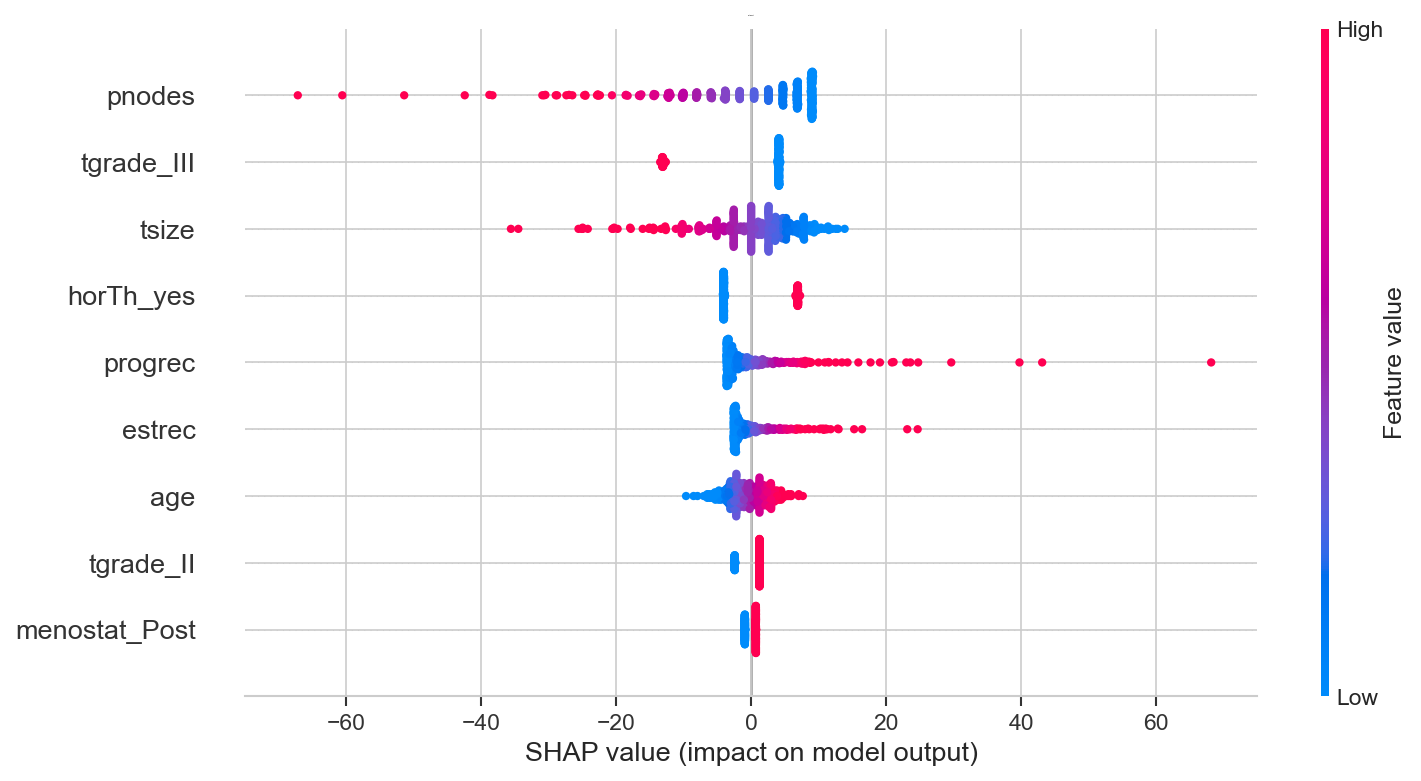

Supplement: Multimedia Appendix 5 [file ai_v3i1e47652_app5.zip › Publish/gbsg2/smpc-analysis_dot.png]

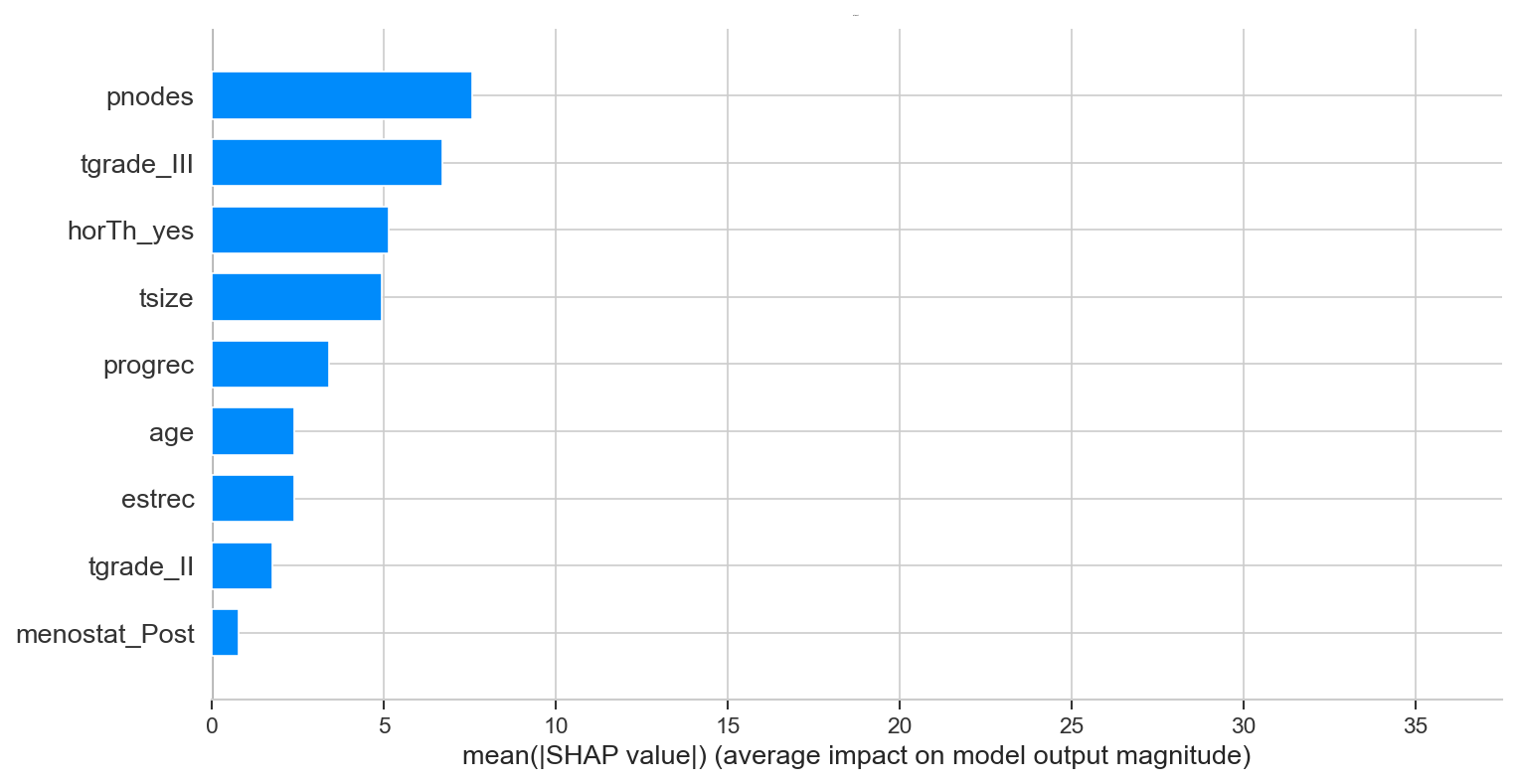

Supplement: Multimedia Appendix 5 [file ai_v3i1e47652_app5.zip › Publish/gbsg2/federated-analysis_bar.png]
